# Predictive Models for Long Term Survival of AML Patients Treated with Venetoclax and Azacitidine or 7+3 Based on Post Treatment Events and Responses: Retrospective Cohort Study

**DOI:** 10.2196/54740

**Published:** 2024-08-21

**Authors:** Nazmul Islam, Jamie S Reuben, Justin Dale, James W Coates, Karan Sapiah, Frank R Markson, Craig T Jordan, Clay Smith

**Affiliations:** 1 RefinedScience Aurora, CO United States; 2 Division of Hematology University of Colorado Anschutz Aurora, CO United States; 3 Department of Medicine University of Colorado Anschutz Aurora, CO United States

**Keywords:** Leukemia, Myeloid, Acute, Venetoclax, Azacitidine, Anthracycline, Arabinoside, Cytosine, Clinical Decision Support, Clinical Informatics, Machine Learning, Predictive Model, Overall Survival

## Abstract

**Background:**

The treatment of acute myeloid leukemia (AML) in older or unfit patients typically involves a regimen of venetoclax plus azacitidine (ven/aza). Toxicity and treatment responses are highly variable following treatment initiation and clinical decision-making continually evolves in response to these as treatment progresses. To improve clinical decision support (CDS) following treatment initiation, predictive models based on evolving and dynamic toxicities, disease responses, and other features should be developed.

**Objective:**

This study aims to generate machine learning (ML)–based predictive models that incorporate individual predictors of overall survival (OS) for patients with AML, based on clinical events occurring after the initiation of ven/aza or 7+3 regimen.

**Methods:**

Data from 221 patients with AML, who received either the ven/aza (n=101 patients) or 7+3 regimen (n=120 patients) as their initial induction therapy, were retrospectively analyzed. We performed stratified univariate and multivariate analyses to quantify the association between toxicities, hospital events, and short-term disease responses and OS for the 7+3 and ven/aza subgroups separately. We compared the estimates of confounders to assess potential effect modifications by treatment. 17 ML-based predictive models were developed. The optimal predictive models were selected based on their predictability and discriminability using cross-validation. Uncertainty in the estimation was assessed through bootstrapping.

**Results:**

The cumulative incidence of posttreatment toxicities varies between the ven/aza and 7+3 regimen. A variety of laboratory features and clinical events during the first 30 days were differentially associated with OS for the two treatments. An initial transfer to intensive care unit (ICU) worsened OS for 7+3 patients (aHR 1.18, 95% CI 1.10-1.28), while ICU readmission adversely affected OS for those on ven/aza (aHR 1.24, 95% CI 1.12-1.37). At the initial follow-up, achieving a morphologic leukemia free state (MLFS) did not affect OS for ven/aza (aHR 0.99, 95% CI 0.94-1.05), but worsened OS following 7+3 (aHR 1.16, 95% CI 1.01-1.31) compared to that of complete remission (CR). Having blasts over 5% at the initial follow-up negatively impacted OS for both 7+3 (*P*<.001) and ven/aza (*P*<.001) treated patients. A best response of CR and CR with incomplete recovery (CRi) was superior to MLFS and refractory disease after ven/aza (*P*<.001), whereas for 7+3, CR was superior to CRi, MLFS, and refractory disease (*P*<.001), indicating unequal outcomes. Treatment-specific predictive models, trained on 120 7+3 and 101 ven/aza patients using over 114 features, achieved survival AUCs over 0.70.

**Conclusions:**

Our findings indicate that toxicities, clinical events, and responses evolve differently in patients receiving ven/aza compared with that of 7+3 regimen. ML-based predictive models were shown to be a feasible strategy for CDS in both forms of AML treatment. If validated with larger and more diverse data sets, these findings could offer valuable insights for developing AML-CDS tools that leverage posttreatment clinical data.

## Introduction

Acute myeloid leukemia (AML) is an aggressive malignancy of the myeloid cells in the hematopoietic system [1]. Without treatment, patients can die within days to months due to infection, bleeding, organ damage, or other complications. The treatment approaches for AML vary significantly based on the patient’s ability or willingness to tolerate intensive therapy [1,2]. For young and fit patients, a typical intensive therapy approach involves induction treatment with anthracycline and cytosine arabinoside, commonly known as 7+3 therapy. This is followed by additional consolidative chemotherapy or an allogeneic stem cell transplantation (alloSCT), depending on the genetic features of the AML at diagnosis, as well as the clinical status of the patient and the AML after induction therapy [3,4]. This intensive approach is potentially curative but is associated with high morbidity, mortality, cost, and prolonged hospital stays. For patients who are not suitable for, or choose to decline, this intensive approach due to age, fitness, or personal preference at diagnosis, the Bcl-2 inhibitor venetoclax, in combination with a hypomethylating agent such as azacitidine or decitabine, has become a new standard of care [5-7]. This strategy is typically aimed at prolonging life rather than achieving a cure and is associated with less morbidity, treatment-related mortality, and time spent in the hospital compared with intensive approaches [8].

We and others have described a variety of features of both patients and AML at diagnosis that are associated with long-term survival and other outcomes following treatment with either intensive approaches or venetoclax plus azacitidine (ven/aza)–based treatments [9-11]. However, the treatment course for patients with AML is highly variable, and factors such as “fitness” can change significantly, for better or worse, during treatment. Additionally, there is significant variability in AML responses to therapy during treatment, which are difficult to predict at diagnosis. As a result, prognosis and clinical decision-making can evolve significantly based on events and responses occurring after the initiation of treatment. Therefore, identifying key prognostic features that develop following treatment and are associated with long-term disease behavior and survival is essential for refining clinical decision-making over time. For intensive treatment approaches, events such as the achievement of a morphologic complete remission (CR), the presence or absence of minimal residual disease (MRD) detected by flow cytometry or next-generation sequencing, and other AML-related assessments that occur following the initiation of therapy are predictive of long-term outcomes [12-28]. Many of these early response indicators are useful for guiding subsequent therapeutic decisions. For example, the presence of MRD after induction therapy with 7+3 or other intensive treatments can predict the success of alloSCT, guide the choice of transplant type, and identify high-risk patients who may benefit from post-transplant maintenance therapy [16,18-20,29-33]. In ven/aza treatment, achieving MRD negativity is associated with improved event-free survival and overall survival (OS) [34]. However, in contrast to intensive approaches, there is limited knowledge about how toxicities, early clinical events, and short-term treatment responses are associated with disease behavior and long-term patient outcomes with this therapy.

To address this gap, we evaluated clinical events, toxicities, short-term outcomes, biomarkers, and other features occurring after the initiation of treatment with either 7+3 or ven/aza to understand their association with OS. Additionally, we developed models to assess the long-term dynamic behavior of responses to 7+3 and ven/aza based on short-term disease responses. These studies reveal substantial differences in the clinical and AML features that evolve with the 2 different treatments and highlight how these differences impact prognosis and clinical decision-making.

## Methods

### Patient Populations

Adult, newly diagnosed AML patients who received initial induction therapy with either the ven/aza regimen or the 7+3 regimen at the University of Colorado Hospital (UCH) between January 1, 2013, and December 31, 2020, were included in the study. Patients with acute promyelocytic leukemia and those who voluntarily withdrew within less than 28 days of treatment were excluded. Patient baseline characteristics are summarized in Table S1 in Multimedia Appendix 1. Note that this patient cohort is a subset of the analytical data set as previously described [10]. For exploratory analyses, 120 patients treated with 7+3 and 101 patients treated with ven/aza were included (Figure S1 in Multimedia Appendix 1). Best response analyses were based on 118 out of 120 (98.3%) of the 7+3 patients and all (101/101, 100%) of the ven/aza patients (including those who died before response assessment). For the multistate transition analyses, 115 out of 120 (95.8%) of the 7+3 patients and 98 out of 101 (97.0%) of the ven/aza patients had sufficient data after excluding those without at least one response assessment or who died before their first response assessment. Additionally, 111 out of 120 (92.5%) of the 7+3 patients and 91 out of 101 (90.1%) of the ven/aza patients had 30-day follow-up data adequate for developing machine learning (ML) models.

### Ethical Considerations

This study was a retrospective analysis utilizing a limited data set. A full waiver of consent and a full waiver of Health Insurance Portability and Accountability Act (HIPAA) authorization were granted by the Colorado Multiple Institutional Review Board (approval number 18-1861). The limited data set was securely stored on a HIPAA-compliant, cloud-based data platform, and accessible only to members of the study team.

### Outcome Definitions

Treatment responses, including CR, CR with incomplete hematologic recovery (CRi), morphologic leukemia-free state (MLFS), progressive disease, and stable disease, were defined according to the standard 2017 European LeukemiaNet (ELN) criteria [35]. A patient was classified as “refractory” if the disease persisted after 90 days from the start of treatment or if the disease worsened or showed no improvement at any point during the treatment cycle. Toxicity variables were graded according to the National Comprehensive Cancer Network (NCCN) Common Terminology Criteria for Adverse Events (CTCAE) guidelines [35-37]. Ejection fraction toxicity was defined as detailed in Table S10 in Multimedia Appendix 1. “Induction events” occurred during the initial treatment hospitalization. The “Day_15-55_” disease assessment refers to patient examinations, laboratory analyses, and bone marrow biopsy (BMB) analyses performed closest to day 30 after the initiation of treatment, but within days 15-55 to accommodate variations in assessment timing. “Day 30 readmission events” were defined as clinical events that occurred at least one day after a patient’s discharge from the initial treatment hospitalization and at least one day before the “Day_15-55_” BMB.

### Statistical Learning

Structured and unstructured electronic medical record data were integrated into a heme data mart on the Google Cloud Platform (Alphabet Inc.), as previously described [10]. Descriptive summary statistics of confounders were provided for both 7+3 and ven/aza treatments. Systematic differences between treatments were compared using the Mann-Whitney *U* test, Fisher exact test (for small sample sizes), chi-square test, and standardized mean differences (SMDs). Kaplan-Meier analyses were performed for OS with 95% CIs, assuming right censoring. *P* values for testing the equality of survival curves were reported using log-rank (LR), Tarone-Ware (TW), and Fleming-Harrington (FH) methods. All hypotheses were 2-sided. Cumulative incidence functions (CIFs) for hazards were reported for toxicity along with 95% CIs, LR-based *P* values, and median time to reach the worst toxicity grading from baseline. Multistate survival analyses were conducted using follow-up BMB responses. Occupation probabilities of disease states were estimated using the Aalen-Johansen estimation technique [38-40]. Transition probabilities for moving from one disease state to another over time were estimated assuming a Markov process, with standard errors reported using bootstrap methods across 300 runs [41]. Multivariable Cox proportional hazards (Cox-PH) models with a ridge penalty (ie, L2 norm penalty) were fitted to adjust for multicollinearity, and estimates of adjusted hazard ratios (aHRs) were reported [42]. Before fitting multivariable models, numeric variables were categorized based on clinically meaningful thresholds to enhance interpretability. Noise variables were filtered out using a univariate approach based on accelerated failure time (AFT) models. Tuning parameters for the ridge penalty were selected using the 10-fold cross-validation (CV) approach. Bias-corrected 95% CIs for aHRs were constructed using the fractional random weight bootstrap method with 2000 runs, where weights were computed from a univariate Dirichlet distribution [43].

### Development and Validation of Prognosis Models

**Figure 1:**
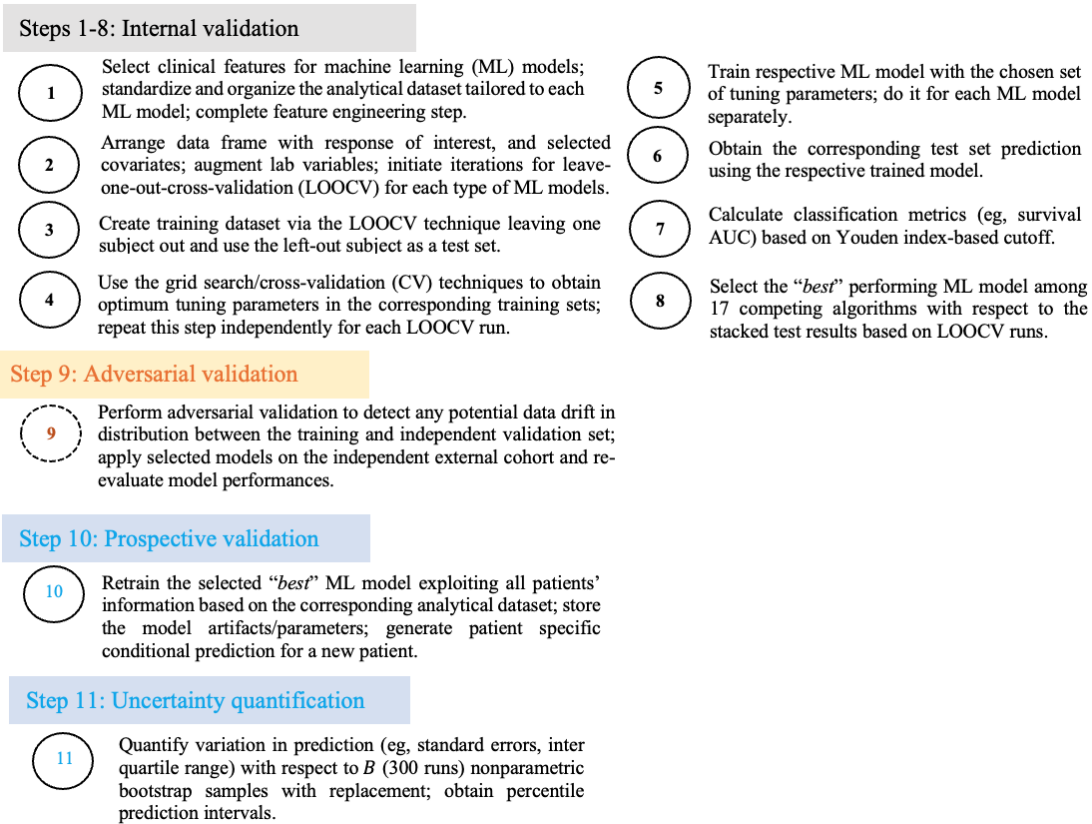
ML architecture. Notation and description of 11 steps for the development of ML models, optimum model selection, validation, prediction, and uncertainty quantification for a newly diagnosed patient with AML. AML: acute myeloid leukemia; AUC: area under the curve.

**Figure 2:**
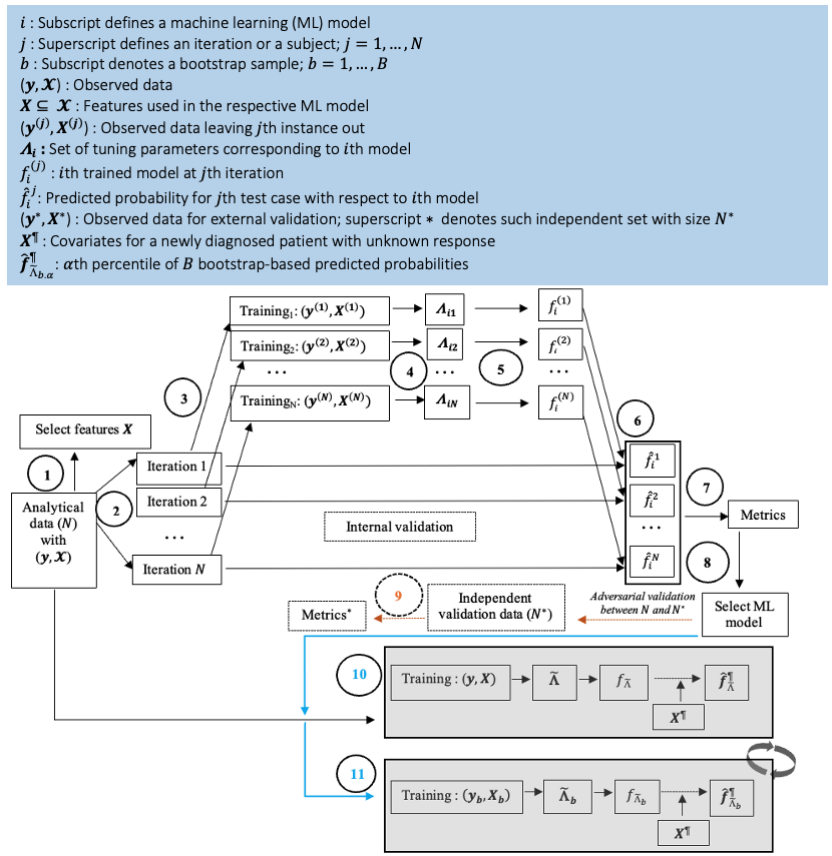
The processes for development of models, optimum model selection, validation, prediction, and uncertainty quantification for a newly diagnosed patient with AML. AML: acute myeloid leukemia; ML: machine learning.

The steps for training and evaluating ML models are depicted in Figures 1 and 2. The process consists of 2 stages. First, internal validation based on CV (steps 1-8) was conducted to select the appropriate ML model for each treatment separately. Second, subject-specific OS predictions, conditional on observed covariates, and the corresponding uncertainty quantification were performed using the selected treatment-specific ML models (steps 10-11). A total of 17 different models ranging from statistical learning-, ML-, and deep learning (DL)–based survival models were used to assess long-term outcomes. These included ensemble-based methods such as the random survival forest (RSF) [44], survival forest with bagging, and conditional inference survival forest [45], as well as Cox-PH models with boosting, penalized Cox-PH models, and parametric AFT models [46] with exponential, Weibull, and log-logistic error structures. These models generated OS probabilities by leveraging over 114 features, as highlighted in Table S12 in Multimedia Appendix 1. The prognostic variable list was further enhanced by creating binary variables based on the first and fifth quintiles of numeric laboratory variables. Regularization penalties [42,47-50] were applied to reduce the risk of overfitting. The penalty terms included ridge, LASSO, elastic-net (eNet), smoothly clipped absolute deviation (SCAD), minimax concave penalty (MCP), adaptive SCAD, adaptive MCP, adaptive eNet, adaptive MCP with L2 norm penalty (mNet), and adaptive SCAD with L2 norm penalty (sNet) [47]. Adaptive models were fitted in 2 stages: in the first stage, models were fitted with ridge penalties, and in the second stage, models were refitted with covariate weights calculated as the reciprocal of parameter estimates from the first stage. Additionally, DL-based survival models (Deep-Surv [51], Deep-LogHaz [52], and Deep-Hit [53]) with 2 hidden layers exploiting neural network structures were used. Tuning parameters for the ensemble-based approaches and DL models were selected using a combination of grid search and CV. For Cox-PH models, regularization penalties were selected using 10-fold CV. Parametric AFT models were fitted with a reduced set of variables. To minimize dimensionality and avoid collinearity in AFT models, a univariate filtering approach was applied, where only variables with Bonferroni-corrected *P* values below a prespecified threshold of 0.20 were included in the final multivariable AFT models. For additional technical details, please refer to Multimedia Appendix 1.

Internal validation was conducted using leave-one-out cross-validation, focusing on several metrics: dynamic area under the curve (AUC) of cumulative case dynamic control of receiver operative characteristics (ROC) curves (cAUC), incident case dynamic control ROC (iAUC) curves, integrated Brier scores, and time-dependent concordance (C) index and Brier score at 1-year survival (denoted by *C_t_* and Brier*_t_*, respectively). The median (M) of cAUCs and iAUCs over event times within 2 years were reported. The model demonstrating the best numerical performance during the internal validation step was retrained using the full data set with appropriately selected tuning parameters. These models were then further evaluated on 2 independent validation sets: 1 for each treatment arm (7+3, n_1_=14; ven/aza, n_2_=30 patients with AML) treated at the University of Colorado Hospital. Adversarial validation, utilizing a generalized linear model with a logit link function, was used to assess potential data drift between the training and validation sets. SMDs were computed, and the predictive performance of the models on the validation sets was reported. For out-of-sample patients, predicted probabilities were reported along with 95% percentile-based confidence bands, derived from 300 nonparametric bootstrap runs. As the primary aim of the study was to develop treatment-specific prognostic models, we did not apply multiple testing corrections for type I errors.

## Results

### Statistical Learning–Based Comparison of Ven/Aza and 7+3 During the First 30 Days of Treatment

Summary statistics for the 7+3 and ven/aza cohorts are presented in Tables S1-S5 in Multimedia Appendix 1. Ven/aza patients were older (median age 72 years, IQR 66-78 years; range 22-90 years) and had more comorbidities and high-risk AML features compared with 7+3 patients (median age 53 years, IQR 41-59 years; range 20-75 years), as previously described [54]. The ven/aza cohort had a higher prevalence of patients with an Eastern Cooperative Oncology Group score of 2 (15/59, 25%) compared with the 7+3 cohort (1/31, 3%). Various diagnostic criteria, including demographic features, comorbidities, laboratory values, and AML pathology characteristics, were associated with OS for both the ven/aza cohort (Figure S2 in Multimedia Appendix 1) and the 7+3 cohort (Figure S4 in Multimedia Appendix 1, “Diagnostic criteria”), consistent with findings described previously [10]. Specific covariates showing notable negative associations (aHR>1) for the ven/aza cohort included prior myelodysplastic syndrome (aHR 1.09, 95% CI 1.03-1.16), prior coagulopathy (aHR 1.12, 95% CI 1.05-1.20), abnormal white blood cell (WBC) count (aHR 1.05, 95% CI 1.00-1.10), blasts >20% (aHR 1.13, 95% CI 1.06-1.22), abnormal platelet count (aHR 1.09, 95% CI 1.04-1.14), elevated uric acid (aHR 1.10, 95% CI 1.03-1.18), high lactate dehydrogenase (aHR 1.14, 95% CI 1.09-1.19), poor cytogenetic risk (aHR 1.12, 95% CI 1.07-1.17), flow cytometry–based CD7 expression (aHR 1.21, 95% CI 1.14-1.28), CD34 expression (aHR 1.06, 95% CI 1.01-1.11), CD38 expression (aHR 1.08, 95% CI 1.02-1.14), and CD11b expression (aHR 1.07, 95% CI 1.01-1.12). Multivariable analyses for the 7+3 cohort revealed similar effects in terms of direction for abnormal WBC, platelet count, uric acid, creatinine, lactate dehydrogenase, poor cytogenetic risk, myeloperoxidase (MPO), and isocitrate dehydrogenase 2 (IDH2). However, more pronounced adverse effects were observed for the ELN-2017–based adverse risk subgroup (aHR 1.06, 95% CI 1.01-1.12), *EGR1* mutation (aHR 1.14, 95% CI 1.07-1.22), and runt-related transcription factor (RUNX; aHR 1.07, 95% CI 1.01-1.13). For more details, see Figures S2 and S4 in Multimedia Appendix 1. The direction of effects was reversed for *CBFB* and *NPM1* between the ven/aza and 7+3 treatment cohorts (Table S13 in Multimedia Appendix 1). Variables indicating genetic abnormalities are detailed in Table S11 in Multimedia Appendix 1.

To determine whether features occurring after diagnosis and the initiation of treatment influenced long-term outcomes, we evaluated the associations between OS and factors such as toxicities, hospital events, transfusions, and short-term disease responses for both treatments separately. A summary of CTCAE toxicities, transfusions, and hospital events, including intensive care unit (ICU) transfers and readmission instances for the 2 treatment cohorts, is provided in Table S2 in Multimedia Appendix 1.

**Figure 3:**
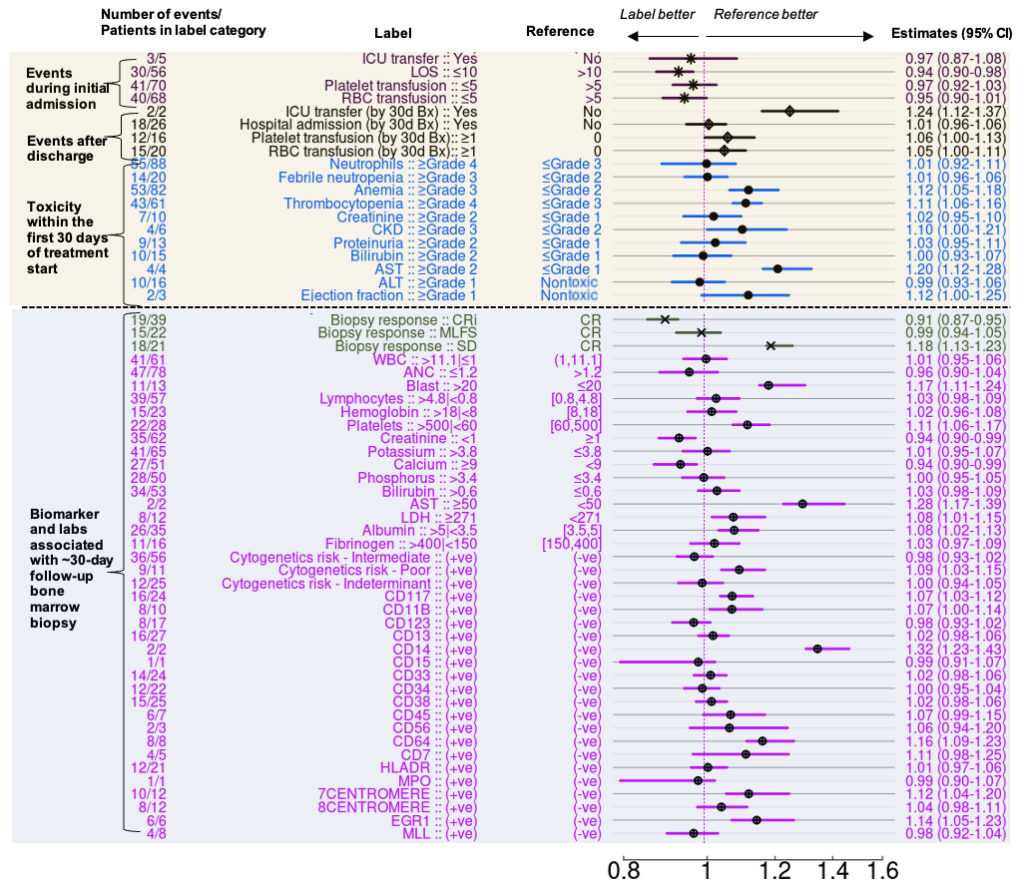
Adjusted hazard ratios (aHRs) for predictors of overall survival for the ven/aza cohort corresponding to events occurring during the first ~30 days of therapy. Reported are the aHRs (vertical tick) and bootstrap-based 95% CIs (horizontal line). “Reference features” correlating with a better outcome are to the right and “Label features” with a better outcome are to the left. The number of patients who died relative to the subset of patients with each feature is summarized at the far left. The table includes findings during the first 30 days and outcomes at the Day_15-55_ bone marrow biopsy assessment at the bottom. Day_15-55_ is defined as the day (or days) between 15 and 55 days from the initiation of treatment when bone marrow biopsy, blood test, and clinical evaluation are conducted to assess response. Different symbols for aHRs were used to differentiate the values between different types of variables. ALT: alkaline phosphatase; ANC: absolute neutrophil count; AST: aspartate transaminase; CKD: chronic kidney disease; CR: complete remission; CRi: complete remission with incomplete hematologic recovery; ICU: intensive care unit; LDH: lactate dehydrogenase; LOS: length of stay; MLFS: morphologic leukemia-free state; RBC: red blood cell; SD: stable disease; ven/aza: venetoclax plus azacitidine; WBC: white blood cell.

For toxicities occurring after the initiation of treatment, grade ≥3 anemia (aHR 1.12, 95% CI 1.05-1.18) and grade ≥4 thrombocytopenia (aHR 1.11, 95% CI 1.06-1.16) were associated with worse OS in the ven/aza group, as observed in both multivariable and univariate analyses (Figure 3, “Toxicity within the first 30 days of treatment”; Figure S3 in Multimedia Appendix 1). Elevated aspartate aminotransferase was also linked to worse OS (aHR 1.20, 95% CI 1.12-1.28) in the ven/aza group (Figure 3), but this association was not found in the 7+3 group, according to both univariate and multivariable analyses (Figures S4 and 5A in Multimedia Appendix 1). Creatinine grade ≥2 in the first 30 days of treatment was associated with worse OS in the 7+3 group, with an aHR of 1.10 (95% CI 1.01-1.20), as seen in both multivariable (Figure S4 in Multimedia Appendix 1, “Toxicity within the first 30 days of treatment start”) and univariate analyses (Figure S5B in Multimedia Appendix 1). By contrast, this association appeared weaker in the ven/aza group (Figure 3, “Toxicity within the first 30 days of treatment start”; Figure S5B in Multimedia Appendix 1). Despite this, worse OS was linked to chronic kidney disease (CKD) grade ≥3 in the ven/aza group, with a multivariable model–based aHR of 1.10 (95% CI 1.00-1.21; Figure 3, “Toxicity within the first 30 days of treatment start”). A similar trend was observed in the 7+3 group (Figure S4 in Multimedia Appendix 1, “Toxicity within the first 30 days of treatment start”). The kinetics of developing CKD differed significantly between ven/aza and 7+3 treatments (Figure S5C in Multimedia Appendix 1). In the ven/aza cohort, CKD was present at diagnosis or developed quickly, with a CIF of approximately 68% at 50 days. By contrast, CKD developed more gradually within the 7+3 cohort, showing a CIF of about 28% at 50 days. There was a trend toward worse outcomes associated with developing ejection fraction toxicity of grade ≥1 for the ven/aza group, although the patient numbers were small (Figure 3, “Toxicity within the first 30 days of treatment start”). For the 7+3 group, no significant association was found between ejection fraction grade ≥1 and OS (aHR 1.02, 95% CI 0.95-1.09; Figure S4 in Multimedia Appendix 1, “Toxicity within the first 30 days of treatment start”). However, both treatment groups developed progressively higher proportions of patients with ejection fraction toxicity grade >1 during the first 30 days of treatment and beyond, at roughly equal rates (Figure S5D in Multimedia Appendix 1). Febrile neutropenia, a common complication of AML therapy, did not show a clear association with OS in either treatment cohort. The aHRs were 1.01 (95% CI 0.96-1.06) for the ven/aza group and 1.02 (95% CI 0.95-1.09) for the 7+3 cohort, indicating no significant effect either by multivariable analysis (Figure 3, “Toxicity within the first 30 days of treatment start” and Figure S4 in Multimedia Appendix 1, “Toxicity within the first 30 days of treatment start”), or by univariate analysis (Figure S5E in Multimedia Appendix 1). Interestingly, for the 7+3 regimen, grade ≥4 neutrophils (CIF ~100% at 50 days) and grade ≥3 febrile neutropenia (CIF ~75% at 50 days) occurred at high levels. By contrast, for the ven/aza cohort, there was a much lower rate of febrile neutropenia (CIF ~25% at 50 days) over time, despite a nearly universal incidence of neurophils (Figure S5F in Multimedia Appendix 1).

For transfusions occurring after the initiation of treatment, Kaplan-Meier analysis revealed that a higher number of platelet and red blood cell transfusions were associated with poorer outcomes in the 7+3 group. This association was evident in both univariate (Figure S5G and S5H in Multimedia Appendix 1) and multivariable analyses (Figure S4 in Multimedia Appendix 1, “Toxicity within the first 30 days of treatment start” and Figure 3, “Toxicity within the first 30 days of treatment start”). Specifically, in the 7+3 cohort, the corresponding aHR indicated negative association with more than 5 platelet transfusions (aHR 1.11, 95% CI 1.06-1.18). This association was less pronounced in the ven/aza group. For hospital events occurring after the initiation of treatment, ICU transfer during the induction period was a particularly poor prognostic feature for patients receiving the 7+3 treatment, with an aHR of 1.18 (95% CI 1.10-1.28) indicating worse outcomes (Figure S4 in Multimedia Appendix 1, “Events during initial admission,” and Figure S5I in Multimedia Appendix 1). By contrast, there was no significant association between ICU transfer following ven/aza treatment and OS during the initial admission (Figure 3, “Events during initial admission”). However, if a patient was discharged and then readmitted to the hospital within the first month of treatment, ICU admission during the readmission was a poor prognostic feature for those treated with ven/aza, with an aHR of 1.24 (95% CI 1.12-1.37; Figure 3, “events after discharge”). For the 7+3 group, initial admissions lasting more than 35 days were associated with worse outcomes (aHR 1.11, 95% CI 1.04-1.18; Figure S4 in Multimedia Appendix 1, “Events during initial admission”). Similarly, for the ven/aza cohort, admissions lasting more than 10 days were associated with poorer outcomes (aHR 1.06, 95% CI 1.02-1.11; Figure 3, “Events during initial admission”).

Next, associations between OS and patient assessments around day 30 (ie, Day_15-55_) after treatment initiation were analyzed. Tables S3-S5 in Multimedia Appendix 1 provide a summary of follow-up patient laboratory values, biomarkers, and AML responses assessed at Day_15-55_ for both ven/aza- and 7+3-treated patients, respectively. At the Day_15-55_ assessment, the ven/aza cohort exhibited lower levels of alanine aminotransferase, aspartate aminotransferase, neutrophils, fibrinogen, lymphocytes, and WBC compared with that of the 7+3 cohort, with SMDs greater than 0.40 (Table S3 in Multimedia Appendix 1). Platelets and hemoglobin levels were also lower in the ven/aza cohort at the Day_15-55_ assessment, but these differences were clinically inconsequential. Summary statistics for AML-related responses are provided in Table S5 in Multimedia Appendix 1. Notably, a lower proportion of ven/aza patients achieved CR at Day_15-55_ (61/111, 55% for 7+3 vs 9/91, 10% for ven/aza). Conversely, a higher proportion of ven/aza patients were in CRi and MLFS compared with those treated with 7+3 (Figure S3 in Multimedia Appendix 1). Patients who achieved CR or CRi at Day_15-55_ had better outcomes compared with those who did not, with an LR-based *P* value of <.001 (Figure S3 in Multimedia Appendix 1, top panel). This was also true for patients who proceeded to receive an alloSCT, with an LR-based *P* value of <.01 (Figure S3 in Multimedia Appendix 1, middle panel). Ven/aza-treated patients who achieved MLFS at Day_15-55_ did not have worse OS compared with those who achieved CR, with an aHR of 0.99 (95% CI 0.94-1.05). By contrast, MLFS at this time point for patients treated with 7+3 was associated with worse outcomes than CR, with an aHR of 1.16 (95% CI 1.01-1.31). This difference was observed in both univariate (Figure S3 in Multimedia Appendix 1, bottom panel) and multivariable analyses (Figure 3, “Biomarker and labs associated with ~30-day follow-up bone marrow biopsy”, and Figure S4 in Multimedia Appendix 1, “Biomarker and labs associated with ~30-day follow-up bone marrow biopsy”). Findings of persistent leukemia in the marrow as detected by flow cytometry, cytogenetics, or fluorescence in situ hybridization were associated with worse outcomes for both treatment groups according to multivariable analysis (Figure S4 in Multimedia Appendix 1, “Biomarker and labs associated with ~30-day follow-up bone marrow biopsy,” and Figure 3, “Biomarker and labs associated with ~30-day follow-up bone marrow biopsy”). Summary statistics for genetics and phenotypic features are provided in Table S4 in Multimedia Appendix 1. Specific posttreatment covariates in the ven/aza cohort demonstrated substantial negative associations, with aHRs exceeding 1, including CD117 (aHR 1.07, 95% CI 1.03-1.12), CD11B (aHR 1.07, 95% CI 1.00-1.14), CD64 (aHR 1.16, 95% CI 1.09-1.23), 7 centromere (aHR 1.12, 95% CI 1.04-1.20), and EGR1 (aHR 1.14, 95% CI 1.05-1.23; Figure 3). Similarly, for the 7+3 patients’ cohort, posttreatment covariates demonstrated inverse associations with OS, with aHRs exceeding 1. Significant negative associations were observed for cytogenetic poor risk (aHR 1.20, 95% CI 1.10-1.31), indeterminant risk (aHR 1.10, 95% CI 1.03-1.17), 8 centromere (aHR 1.22, 95% CI 1.11-1.35), EGR1 (aHR 1.14, 95% CI 1.06-1.22), and FLT3 (aHR 1.22, 95% CI 1.10-1.34; Figure S4 in Multimedia Appendix 1).

**Figure 4:**
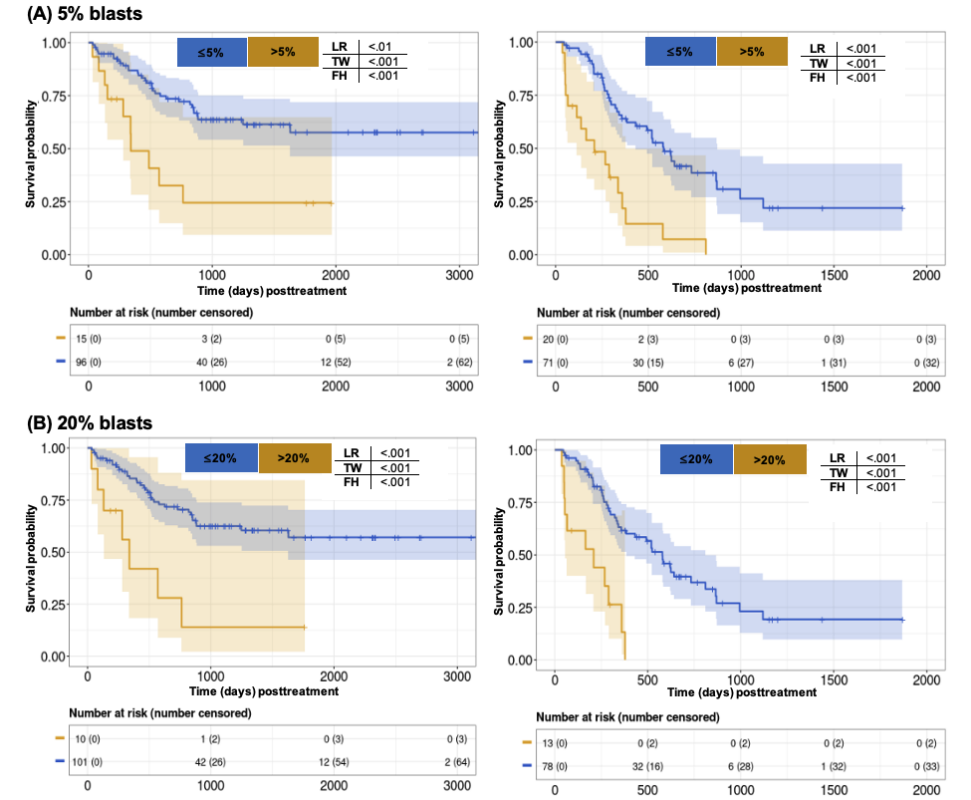
Univariate analysis of blasts recorded at Day_15-55_ response assessment and long-term outcomes (7+3 left, ven/aza right). (A) >5% blasts and outcomes and (B) >20% blasts and outcomes. As described in the "Methods" section, Day_15-55_ is defined as a bone marrow biopsy and other clinical evaluation done within 15-55 days from the initiation of treatment and closest to day 30. *P* values are based on log-rank (LR), Tarone-Ware (TW), and Fleming-Harington (FH) tests. ven/aza: venetoclax plus azacitidine.

For both treatments, the presence of >20% bone marrow blasts and >5% bone marrow blasts at the Day_15-55_ time point were associated with very poor OS by univariate analysis (Figure 4). The corresponding aHRs of >20% blasts were 1.17 (95% CI 1.11-1.24) for the patients treated with ven/aza and 1.11 (95% CI 1.04-1.20) for the patients treated with 7+3. These findings highlight that early toxicities, treatment events, and short-term responses occurring within the first month after treatment initiation are associated with OS for both 7+3 and ven/aza. However, the impact and relevance of these features vary between the 2 treatment regimens.

### Prospective Machine Learning Predictors of OS

To translate the statistical associations between events and responses occurring after the initiation of therapy into predictions that could be potentially applied to individual patients, we developed ML-based predictive models for OS utilizing 17 different ML algorithms based on these baseline and early posttreatment features (Table 1 and Table S12 in Multimedia Appendix 1). The modeling steps are illustrated in Figure 1, and an example of the model development process is shown in Figure 2. Detailed information on feature engineering, model specification, optimization, and final model selection is provided in the section titled “Technical Details” in Multimedia Appendix 1.

**Table 1:** List of machine learning models.

Model abbreviation	Method definition	
RSF	Ensemble survival forest—random	
RSB	Ensemble survival forest—bagging	
Cox-Ridge	Cox regression with ridge penalty	
Cox-LASSO	Cox regression with lasso penalty	
Cox-Relaxed	Cox regression with relaxed lasso penalty	
Cox-Elastic	Cox regression with elastic net penalty	
Cox-adElastic	Cox regression with adaptive elastic net penalty	
Cox-adSCAD	Cox regression with adaptive smoothly clipped absolute deviation (SCAD) penalty	
Cox-adSNET	Cox regression with adaptive SCAD coupled with L2 penalty	
Cox-adMCP	Cox regression with adaptive minimax concave penalty (MCP)	
Cox-adMNET	Cox regression with adaptive MCP coupled with L2 penalty	
Cox-Boost	Boosted Cox regression	
AFT	Accelerated failure time with exponential, Weibull, and log-logistic error	
CISF	Conditional inference survival forest	
Deep-Surv	Cox regression with deep neural net	
Deep-LogHaz	Discrete-time survival estimates by log hazard with neural net	
Deep-Hit	Deep learning–based survival analysis relaxing distributional assumptions	

**Figure 5:**
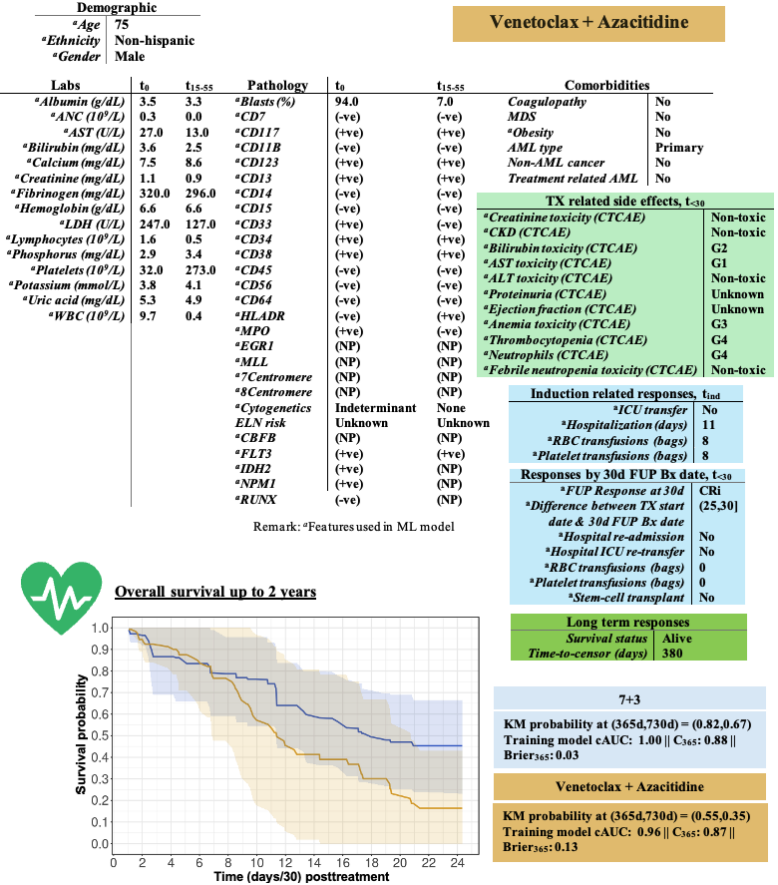
Representative machine learning (ML)-based predictions for a patient chosen randomly from the validation cohort treated with ven/aza. Actual patient values are in the top boxes and the predicted overall survival probabilities along with 95% confidence bands based on the optimal ML models are depicted at the bottom. ALT: alkaline phosphatase; AML: acute myeloid leukemia; ANC: absolute neutrophil count; AST: aspartate transaminase; CKD: chronic kidney disease; CTCAE: Common Terminology Criteria for Adverse Events; FUP: follow-up; ICU: intensive care unit; LDH: lactate dehydrogenase; MDS: myelodysplastic syndrome; RBC: red blood cell; TX: treatment; ven/aza: venetoclax plus azacitidine; WBC: white blood cell.

Among all the models, Cox-Boost (Boosted Cox regression) and RSF achieved median cAUCs of 0.85 (90% CI 0.78-0.88) and 0.80 (90% CI 0.76-0.84) for the ven/aza and 7+3 cohorts, respectively (Tables 2 and 3). In an independent validation set consisting of 16 7+3 and 30 ven/aza patients, median cAUCs of 0.71 and 0.68 were observed for the ven/aza and 7+3 cohorts, respectively (Table 4). DL models resulted in less optimal performance, primarily due to the small sample size and their susceptibility to noise variables. A comparative analysis highlighting the drift between the training and validation cohorts was conducted, with details provided in Tables S6-S9 in Multimedia Appendix 1. These tables cover laboratory values (Table S6 in Multimedia Appendix 1), phenotypic features (Table S7 in Multimedia Appendix 1), genetic biomarkers (Table S8 in Multimedia Appendix 1), and clinical events (Table S9 in Multimedia Appendix 1). For a test patient, the selected ML models were used to generate patient-specific survival probabilities. Figure 5 illustrates the features (top panel) and predicted survival (bottom panel) probabilities for a representative patient randomly selected from the independent validation set. Similarly, subject-specific analyses were conducted for 2 additional patients randomly selected from the internal validation cohorts: 1 treated with ven/aza (Figure S6 in Multimedia Appendix 1) and 1 treated with 7+3 (Figure S7 in Multimedia Appendix 1). The selected models were retrained with 120 7+3 and 100 ven/aza patients for the ven/aza test subject and with 119 7+3 and 101 ven/aza patients for the 7+3 test subject. Although exploratory and limited by sample size, these analyses illustrate that ML predictors of OS can potentially be developed based on clinical events, early disease responses, and biomarkers for both ven/aza and 7+3 treatments. However, as with the statistical analyses, the models that perform optimally are likely to vary between ven/aza and 7+3 treatments. Therefore, they should be developed and validated on a treatment-specific basis.

**Table 2:** Machine learning models of overall survival for ven/aza-treated patients based on events occurring in the first 30 days of treatment and the Day15-55 follow-up assessment.^a^

Overall survival (2 years) prognostic models^b^	Median cAUC^c^ (5th-95th)^d^	Median iAUC^e^ (5th-95th)^d^	C_*t*_^d^	Brier_*t*_^f^	iBrier^f^
RSF	0.79 (0.71-0.86)	0.68 (0.66-0.76)	0.72	0.20	0.17
RSB	0.73 (0.64-0.82)	0.61 (0.60-0.63)	0.67	0.23	0.19
Cox-Ridge	0.82 (0.79-0.89)	0.66 (0.62-0.75)	0.72	0.20	0.17
Cox-LASSO	0.85 (0.78-0.89)	0.69 (0.60-0.72)	0.73	0.20	0.16
Cox-Relaxed	0.83 (0.77-0.88)	0.61 (0.59-0.68)	0.72	0.20	0.17
Cox-Elastic	0.83 (0.78-0.86)	0.63 (0.60-0.70)	0.72	0.20	0.17
Cox-adElastic	0.80 (0.76-0.89)	0.64 (0.60-0.72)	0.66	0.24	0.19
Cox-adSCAD	0.62 (0.58-0.72)	0.52 (0.52-0.53)	0.52	0.29	0.23
Cox-adSNET	0.78 (0.73-0.85)	0.65 (0.62-0.69)	0.66	0.23	0.19
Cox-adMCP	0.62 (0.51-0.66)	0.55 (0.54-0.56)	0.55	0.26	0.22
Cox-adMNET	0.78 (0.73-0.85)	0.65 (0.62-0.69)	0.66	0.23	0.20
Cox-Boost^g^	0.85 (0.78-0.88)	0.66 (0.61-0.74)	0.76	0.19	0.16
AFT-Exponential	≤0.50	0.62 (0.60-0.64)	0.73	0.19	0.16
AFT-Weibull	≤0.50	0.65 (0.64-0.66)	0.72	0.22	0.17
AFT-log-logistic	≤0.50	0.65 (0.64-0.66)	0.75	0.21	0.17
CISF	≤0.50	0.64 (0.61-0.65)	0.58	0.22	0.21
Deep-Surv	≤0.50	0.52 (0.52-0.53)	0.46	0.25	0.44
Deep-Hit	0.52 (≤0.50-0.55)	0.52 (0.51-0.52)	0.38	0.32	0.34
Deep-LogHaz	≤0.50	0.53 (0.53-0.54)	0.46	0.43	0.74

^a^Time-dependent AUCs (ie, median cAUC and iAUC), time-dependent concordance (C) index and Brier score at 1 year, and integrated Brier score were reported. As described in the “Methods” section, Day_15-55_ is defined as a bone marrow biopsy and other clinical evaluation done within 15-55 days from the initiation of treatment.

^b^See Table 1 for models and method definitions.

^c^cAUC: cumulative case dynamic control of receiver operative characteristics.

^d^The higher value (ie, close to 1) means better numerical performance.

^e^iAUC: incident case dynamic control of receiver operative characteristics.

^f^The lower value (ie, close to 0) means better numerical performance.

^g^The selected final model for ven/aza.

**Table 3:** Machine learning models of overall survival for 7+3-treated patients based on events occurring in the first 30 days of treatment and the Day15-55 follow-up assessment.^a^

Overall survival (2 years) prognostic models^b^	Median cAUC^c^ (5th-95th)^d^	Median iAUC^e^ (5th-95th)^d^	C_*t*_^d^	Brier_*t*_^f^	iBrier^f^
RSF^g^	0.80 (0.76-0.84)	0.71 (0.70-0.73)	0.74	0.12	0.16
RSB	0.78 (0.75-0.84)	0.71 (0.71-0.72)	0.73	0.13	0.15
Cox-Ridge	0.80 (0.75-0.86)	0.70 (0.69-0.73)	0.71	0.11	0.17
Cox-LASSO	0.71 (0.67-0.83)	0.64 (0.63-0.65)	0.65	0.13	0.18
Cox-Relaxed	0.68 (0.61-0.73)	0.64 (0.63-0.65)	0.65	0.13	0.19
Cox-Elastic	0.73 (0.69-0.83)	0.65 (0.65-0.66)	0.67	0.12	0.18
Cox-adElastic	0.76 (0.70-0.79)	0.68 (0.67-0.68)	0.70	0.13	0.17
Cox-adSCAD	0.70 (0.44-0.73)	0.66 (0.66-0.67)	0.68	0.14	0.18
Cox-adSNET	0.71 (0.64-0.72)	0.66 (0.66-0.67)	0.68	0.13	0.17
Cox-adMCP	0.61 (0.47-0.65)	0.62 (0.61-0.62)	0.64	0.13	0.18
Cox-adMNET	0.71 (0.65-0.73)	0.66 (0.66-0.67)	0.68	0.13	0.17
Cox-Boost	0.70 (0.61-0.79)	0.63 (0.63-0.64)	0.64	0.13	0.18
AFT-Exponential	≤0.50	0.51 (0.50-0.51)	0.60	0.13	0.20
AFT-Weibull	≤0.50	0.53 (0.53-0.54)	0.57	0.14	0.20
AFT-log-logistic	≤0.50	0.57 (0.56-0.57)	0.60	0.14	0.20
CISF	≤0.50	0.63 (0.62-0.64)	0.61	0.13	0.23
Deep-Surv	≤0.50	0.58 (0.58-0.59)	0.39	0.15	0.44
Deep-Hit	0.55 (≤0.50-0.64)	≤0.50	0.53	0.14	0.59
Deep-LogHaz	≤0.50	0.61(0.60,0.61)	0.45	0.48	≥1.00

^a^Time-dependent AUCs (ie, median cAUC and iAUC), time-dependent concordance (C) index and Brier score at 1 year, and integrated Brier score were reported. As described in the “Methods” section, Day_15-55_ is defined as a bone marrow biopsy and other clinical evaluation done within 15-55 days from the initiation of treatment.

^b^See Table 1 for models and method definitions.

^c^cAUC: cumulative case dynamic control of receiver operative characteristics.

^d^The higher value (ie, close to 1) means better numerical performance.

^e^iAUC: incident case dynamic control of receiver operative characteristics.

^f^The lower value (ie, close to 0) means better numerical performance.

^g^Selected model for 7+3.

**Table 4:** Numerical performances of the chosen machine learning models for ven/aza^a^ and 7+3 on independent validation cohorts with respect to overall survival (≤2 years).

Treatment	Machine learning models	Median cAUC^b,c^	Median iAUC^b,d^	C_*t*_^b^	Brier_*t*_^e^	iBrier^e^
7+3 (n=14)	RSF^f^	0.71	0.69	0.66	0.19	0.21
ven/aza (n=30)	Cox-Boost^f^	0.68	0.64	0.66	0.29	0.23

^a^ven/aza: venetoclax plus azacitidine.

^b^The higher value (ie, close to 1) means better numerical performance.

^c^cAUC: cumulative case dynamic control of receiver operative characteristics.

^d^iAUC: incident case dynamic control of receiver operative characteristics.

^e^The lower value (ie, close to 0) means better numerical performance.

^f^See Table 1 for models and method definitions.

### Association Between Events Occurring During the First Year of Therapy and Overall Survival

**Figure 6:**
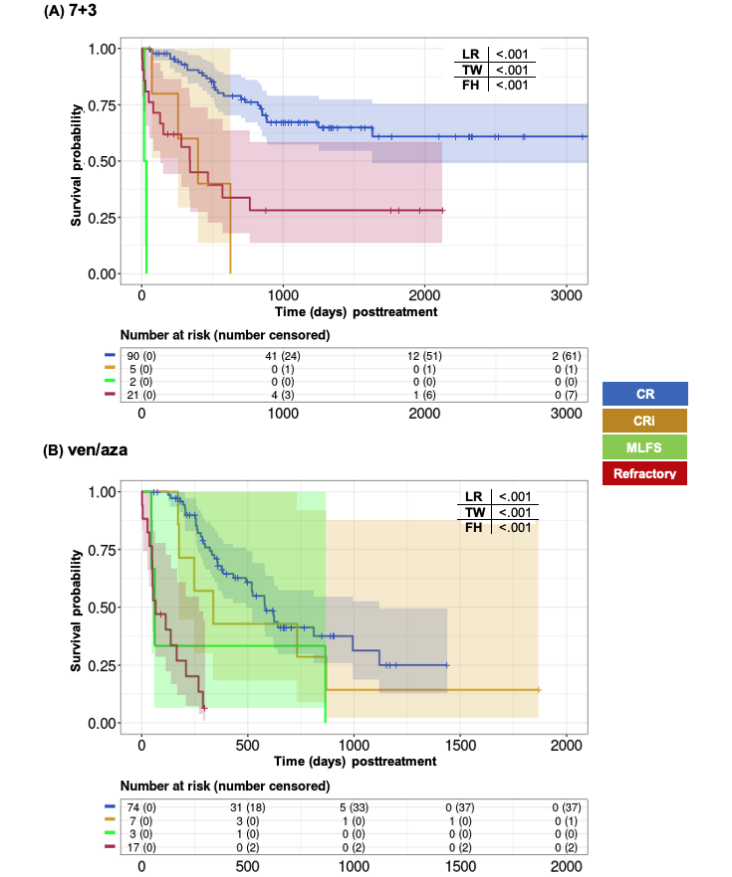
Univariate Kaplan-Meier analysis of best response during the first 180 days' assessment and long-term outcomes with (A) 7+3 and (B) ven/aza.*P* values are based on log-rank (LR), Tarone-Ware (TW), and Fleming-Harington (FH) tests. CR: complete remission; CRi: complete remission with incomplete hematologic recovery; MLFS: morphologic leukemia-free state; ven/aza: venetoclax plus azacitidine.

As treatment events and responses in AML can evolve beyond the first month of treatment, we investigated associations between later disease responses and OS. Initially, we examined the association between the best response after the treatment initiation and long-term OS. Among patients treated with 7+3, achieving CR as the best response correlated with a 4-year OS rate of approximately 60%. Conversely, achieving CRi, MLFS, or being nonresponsive (refractory) as the best responses correlated with an OS rate of approximately 25% or less. An LR–based *P* value <.001 indicated significant differences between survival curves (Figure 6A). In the ven/aza-treated cohort, both CR and CRi were similarly associated with OS, whereas MLFS and nonresponses correlated with lower OS (Figure 6B). It is important to note that Kaplan-Meier survival curves might be influenced by alloSCT. For instance, out of 21 refractory 7+3 patients, 10 (48%) underwent alloSCT, potentially leading to an overestimation of the corresponding survival curve. By contrast, only 3 (18%) out of 17 refractory ven/aza patients underwent alloSCT.

**Figure 7:**
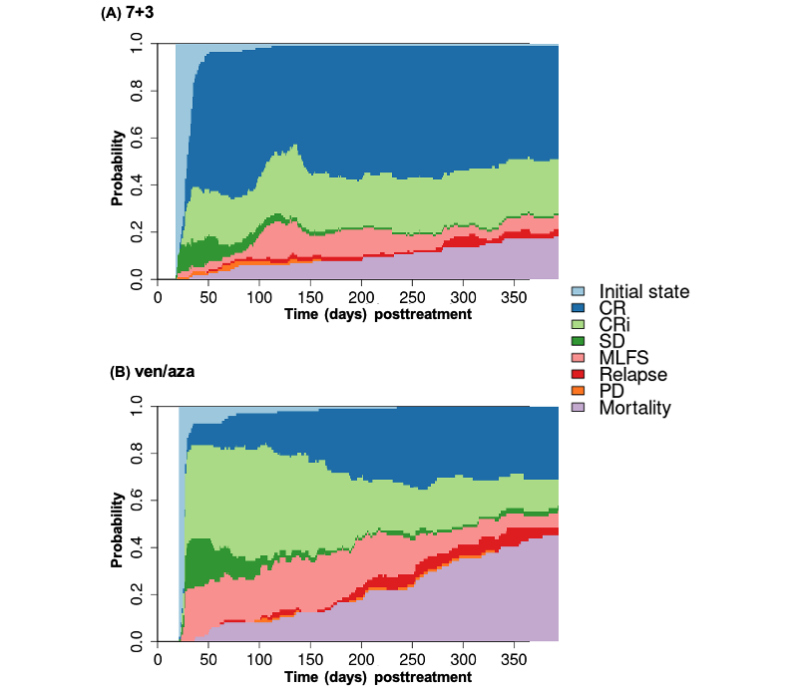
Overall trends in disease state changes during first year of treatment for (A) 7+3 and (B) ven/aza. CR: complete remission; CRi: complete remission with incomplete hematologic recovery; MLFS: morphologic leukemia-free state; PD: progressive disease; SD: stable disease; ven/aza: venetoclax plus azacitidine.

**Figure 8:**
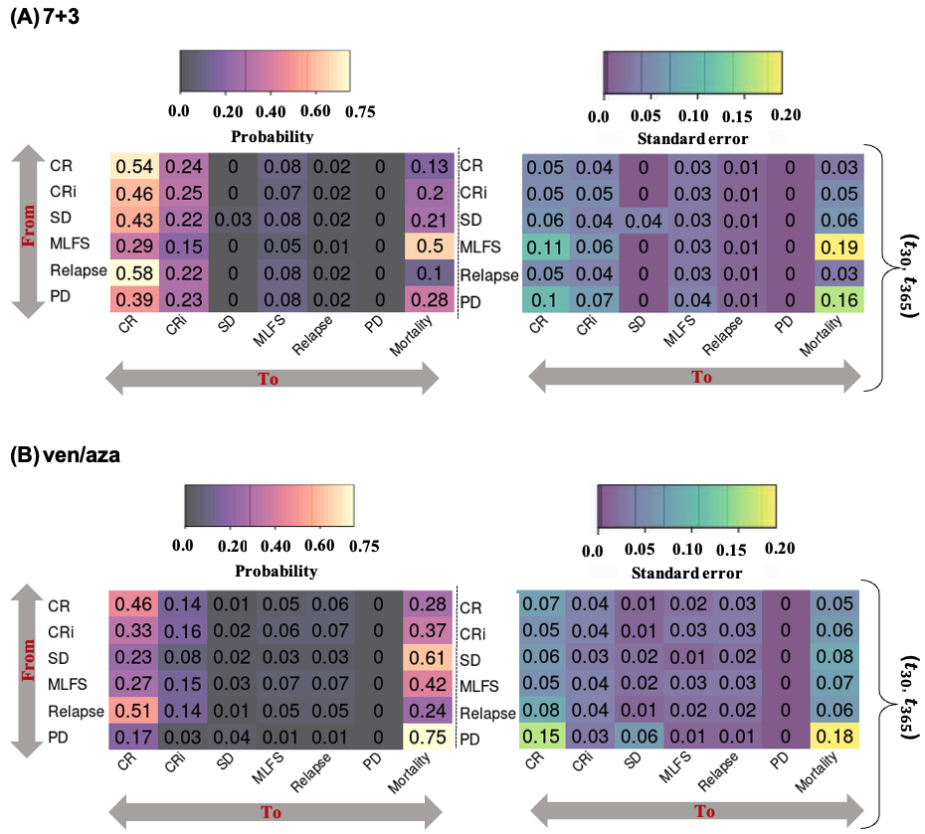
Probabilities of transitions from treatment responses (y-axis) achieved by day 30 of treatment to states (x-axis) within 365 days following treatment for (A) 7+3-treated patients and (B) ven/aza-treated patients. The state transition probabilities are on the left panels and SEs are on the right. CR: complete remission; CRi: complete remission with incomplete hematologic recovery; MLFS: morphologic leukemia-free state; PD: progressive disease; SD: stable disease; ven/aza: venetoclax plus azacitidine.

Next, we examined the kinetics of achieving the best response in the 2 treatment groups. The pattern of reaching the best responses differed between the 7+3 and ven/aza groups (Figure 7), as did the overall frequencies of various treatment response outcomes (Figure S8 in Multimedia Appendix 1). At the population level, the 7+3 cohort quickly reached a relatively stable state by day 30 (Figure 7A). By contrast, the ven/aza cohort showed a continual evolution with conversions from CRi and MLFS to either CR or death (Figure 7B). The disease-state transition probabilities between days 30 and 365 also differed significantly between 7+3 (Figure 8A) and ven/aza (Figure 8B). Achieving CR (0.28; SE 0.05), CRi (0.37; SE 0.06), or MLFS (0.42; SE 0.07) around day 30 after ven/aza treatment showed similar probabilities of transitioning to mortality within a year. By contrast, CR (0.13; SE 0.03) and CRi (0.20; SE 0.05) had comparable transitioning rates to mortality for 7+3. Ven/aza patients with stable disease and progressive disease around day 30 had poorer OS, with 1-year mortality rates of 0.61 (SE 0.08) and 0.75 (SE 0.18), respectively. These observations contrast with that of 7+3, presumably because 7+3 patients were generally fit enough to undergo additional therapies aimed at disease control during this period. Similar observations were noted in disease-state transition probabilities between days 90-365 (Figure S9 in Multimedia Appendix 1) and days 180-365 (Figure S10 in Multimedia Appendix 1). Unlike the 7+3 subgroup, patients treated with ven/aza who achieved any disease state around 180 days had a higher likelihood of transitioning to mortality, with the highest probability observed for the relapse state (0.73; SE 0.16 for ven/aza and 0.54; SE 0.33 for 7+3). Specifically, patients in the MLFS disease state around 180 days transitioned more rapidly to mortality with ven/aza (0.37; SE 0.08) compared with 7+3 (0.14; SE 0.07; refer to Figure S10 in Multimedia Appendix 1). These results further confirm that associations with short- and long-term outcomes differ following ven/aza and 7+3 treatments. The kinetics of responses with ven/aza are notably more dynamic and occur over different time frames compared with those with 7+3. These observations underscore the necessity for distinct response criteria, maintenance strategies, and timing of measurements tailored to each therapy.

## Discussion

### Principal Findings

The main findings of this study indicate that various clinical events occurring during the first month of ven/aza treatment correlate with OS, distinct from outcomes following the 7+3 treatment. Achieving CR/CRi or MLFS around day 30 (ie, Day_15-55_) after ven/aza treatment has a similar long-term prognostic impact, while failure to achieve MLFS around day 30 with 7+3 indicates poorer outcomes. We also identified clinical features such as bone marrow blasts >5%, flow cytometric and genetic detection of AML, and AML-related cytogenetic factors at reassessment as having negative prognostic impacts on OS. Based on these observations, detection of persistent leukemia in the bone marrow around day 30 following ven/aza treatment suggests consideration of alternative therapies. By contrast, achieving CR/CRi/MLFS around day 30 with minimal evidence of persistent leukemia following ven/aza is associated with improved OS, indicating the benefit of continuing this treatment. However, we also found that failure to achieve CR/CRi by approximately day 180 after ven/aza initiation has negative implications for OS. This suggests that alternative therapies should be considered if the milestone of achieving CR/CRi by this time point is not met.

We also found that certain hospital events and toxicities occurring after the initiation of ven/aza treatment have prognostic implications, which differ from those seen with the 7+3 treatment. For instance, ICU admission during the initial ven/aza treatment was not associated with worse outcomes, whereas ICU transfer during the initial hospitalization for 7+3 was a poor prognostic factor. Additionally, grade ≥4 thrombocytopenia and grade ≥3 anemia were more pronounced as poor prognostic indicators for ven/aza compared with 7+3. The incidence of renal impairment was similar for both treatments; however, elevated creatinine, proteinuria, and CKD were associated with worse OS among patients treated with 7+3, but less so among those treated with ven/aza. The progression of grade ≥1 ejection fraction toxicity over time was comparable between both treatment arms. While there is a well-known association between anthracyclines in the 7+3 regimen and cardiac toxicity, such an association has not been previously described for ven/aza. These associative findings, albeit based on small sample sizes, may warrant further investigation.

### Prospective Predictive Models for AML Clinical Decision Support

To translate the statistical associations between events and outcomes following the initiation of AML therapy into patient-specific prognostic models, we developed predictive ML models independently for OS with ven/aza and 7+3 treatments. These models utilized baseline and early disease responses, biomarkers, and clinical events. Several models achieved relatively high AUCs of 0.80 and 0.85 in the internal validation step. However, in the independent validation phase, AUCs were lower at 0.71 and 0.68 for the 7+3 and ven/aza cohorts, respectively. This predictive discrepancy may stem from data drift, yet we tested the models to evaluate their performance on nonhomogeneous data. Although not ideal, we contend that such drifts are typical in real-world data sets. Nevertheless, these findings illustrate the feasibility of developing ML-based individual predictors using patient data that evolve. This capability allows clinical decision-making to adapt to individual changes in treatment side effects and responses. This effort contributes to an expanding body of research utilizing ML to predict outcomes in the treatment of AML and other hematologic malignancies [55-58]. For instance, Park et al [55] evaluated the prognostic performance of ELN genetic risk stratification models using unsupervised ML techniques and found suboptimal predictions for OS in older patients with AML, indicating a need for new risk models in this demographic. Karami et al [56] identified novel prognostic factors for survival in patients with AML, incorporating demographic and AML-specific features through ML approaches. Shaikh et al [57] developed a novel risk stratification model for patients with AML and RUNX1-RUNX1T1 using supervised machine learning models. This model assesses risk based on somatic mutations in *Flt3*, *NRAS*, and other genes. Lastly, Eckardt et al [58] conducted a review of various ML approaches for AML diagnosis, prognosis, and risk stratification, emphasizing their evolving and potentially impactful role in this specific disease area. To our knowledge, no prior predictive ML-based survival models with uncertainty quantification have been developed in the AML literature by exploiting both patient- and event-specific long-term dynamic features at this level of detail.

### Limitations

Our study is limited by relatively small data sets, and our results require validation on larger data sets from diverse centers ensuring heterogeneity. Although we adjusted for high collinearity among variables, missingness, and overfitting, these issues need further careful consideration in larger external data sets. Additionally, our single-center data set consists of half real-world and half clinical trial data, which may potentially bias the results compared with more diverse population-based data sets. Lastly, any comparison between the 2 primary treatments, ven/aza and 7+3, is influenced by differences in patient demographics, comorbidities, and other inherent features. Ven/aza is currently approved only for older and unfit patients, whereas 7+3 is primarily used in younger and fitter patients. Additionally, 24 out of 101 ven/aza patients (23.8%) underwent at least one alloSCT after the initiation of treatment, whereas 79 out of 120 7+3 patients (65.8%) underwent at least one alloSCT. Survival curves in AML are affected by alloSCT, which can significantly impact OS and necessitate adjustments and modifications in ML modeling, a direction we plan to explore in future studies. Because of these complexities, the extent and direction of association with OS for confounding factors vary across the treatments we evaluated. However, our primary objective was not to establish causal treatment effectiveness by treating ven/aza as the treatment group and 7+3 as the control for which a classical propensity score–based or weighted method is recommended to ensure balances in data distributions between the 2 treatment arms. Instead, our primary objective was to separately explore the variations in data to assess whether treatment modifies the effects (ie, directions) of confounders on OS. Therefore, we treated 7+3 and ven/aza as effect modifiers and conducted “stratified” multivariable analyses, creating subgroups of patients treated with 7+3 and ven/aza [59]. This stratification approach minimizes the variation attributed to treatment differences significantly, and by accounting for the same set of potential confounders in both models, it enhances the comparability of results.

### Conclusions

Despite these considerations, our results have highlighted significant clinical implications of posttreatment outcomes, clinical events, and toxicities on long-term outcomes and treatment decisions in AML, demonstrating differences between ven/aza and 7+3. Additionally, these observations suggest strong potential to develop ML-based predictive models which could ultimately offer crucial ongoing clinical decision support for patients and providers as toxicities, responses, and other events evolve dynamically throughout treatment cycles.

## Appendix

Multimedia Appendix 1 Additional pertinent results.

## Abbreviations

AFT: accelerated failure time
aHR: adjusted hazard ratio
alloSCT: allogeneic stem cell transplantation
AML: acute myeloid leukemia
AUC: area under the curve
BMB: bone marrow biopsy
C: concordance
cAUC: cumulative case dynamic control of receiver operative characteristics
CDS: clinical decision support
CIF: cumulative incidence function
CKD: chronic kidney disease
CR: complete remission
CRi: complete remission with incomplete hematologic recovery
CTCAE: Common Terminology Criteria for Adverse Events
CV: cross-validation
DL: deep learning
ELN: European Leukemia Net
eNet: elastic-net
iAUC: incident case dynamic control of receiver operative characteristics
ICU: intensive care unit
LOOCV: leave-one-out-cross-validation
M: median
MCP: minimax concave penalty
ML: machine learning
MLFS: morphologic leukemia-free state
MRD: minimal residual disease
NCCN: National Comprehensive Cancer Network
OS: overall survival
PD: progressive disease
PH: proportional hazard
RBC: red blood cell
ROC: receiver operative characteristic
RSF: random survival forest
RUNX: runt-related transcription factor
SCAD: smoothly clipped absolute deviation
ven/aza: venetoclax plus azacitidine
WBC: white blood cell

## Data Availability

The raw, individual patient data are protected and not available due to data privacy laws. The processed data are available at reasonable request to the corresponding author.
