# Supplementary material for: Predictive Models for Long Term Survival of AML Patients Treated with Venetoclax and Azacitidine or 7+3 Based on Post Treatment Events and Responses: Retrospective Cohort Study
Source: JMIR Cancer. 2024 Aug 21;10:e54740. doi: 10.2196/54740 (PMC11375398; doi:10.2196/54740)
Supplement: Multimedia Appendix 1 [file cancer_v10i1e54740_app1.docx]

SUPPLEMENTAL MATERIALS

for

**Predictive Models for Long Term Survival of AML Patients Treated with Venetoclax and Azacitidine or 7+3 Based on Post Treatment Events and Responses: Retrospective Cohort Study**

**­­**

Nazmul Islam, MS, MBA, PhD

Jamie S. Reuben, MPH, MPPA, BS

Justin Dale, BBA

James W. Coates, BS, MS

Karan Sapiah, MS

Frank R. Markson, BS

Craig T. Jordan, PhD

Clay Smith, MD

From RefinedScience (NI, JSR, JD, JWC, KS, FRM), Aurora, Colorado, USA; Division of Hematology (CTJ), University of Colorado Anschutz Medical Campus, Aurora, Colorado, USA;

Department of Medicine (CS), University of Colorado Anschutz Campus, Aurora, Colorado, USA.

# Technical details

## Feature selection

Feature selection step corresponds to Figure 3 Step – 1. A univariable association filter approach was used to screen for potential risk factors at diagnosis. Any variable having a significant univariate test by accelerated failure time model at a prespecified threshold of 0.25 is considered as a potential predictor for statistical learning models. In addition, variables selected at diagnosis or treatment start date (i.e., Day 0) were added in the model along with the corresponding values recorded at ~day 30 (i.e. Day_15-55_  at which the first follow-up bone marrow biopsy responses were recorded) as separate covariates. Two types of features were added in the predictive machine and deep learning models: patient-specific and event-specific. Patient-specific variables were comprised of basic demographic variables, common labs, phenotypic variables, cytogenetic risk, and AML related mutation. Events-specific variables consisted of AML treatment specific events occurred during initial hospitalization, events after discharge, and toxicities recorded within the first 30 days of treatment start date. The variables included total length of stay, intensive care unit transfers, transfusions, follow-up responses, and CTCAE defined toxicities. We exploited 114 features (without engineered features) to develop ML and DL based predictive models for two strata (i.e., 7+3 and ven/aza); for details of the list, we refer to Supplemental Table 12. While we developed models independently for each stratum, we accounted for the same set of potential confounding variables across populations to increase the comparability of results.

## Feature engineering

Feature augmentation step corresponds to Figure 3 Step : 2. Feature engineering was performed to augment numerical performances of the ML models by adding supplemental variables within each stratum (i.e., 7+3 and ven/aza) separately. For each numeric lab variable, two additional binary variables were created with respect to the first and fifth quintiles of the corresponding lab variables. For consistency, all numeric variables are scaled by Min-Max standardization. Numeric variables in the test set are scaled with respect to the minimum and maximum values of the corresponding training data set to minimize potential data leakage. For missing uric-acid, LDH, ANC, lymphocytes, and phosphorus, we imputed values based on the corresponding median values of the analytical dataset. For categorical missing data, we generated a separate category as “missing” for each corresponding feature and subsequently added in the respective model to retain the information that certain data point is missing while adjusting for missingness with ease.

## Model specification

Model specification step corresponds to Figure 3 Step: 3. We have fitted 19 different predictive models where the complexity, model specifications, tuning parameters, and functional forms for each model vary significantly. In general, penalized Cox regression models account only for the main effects and require a few tuning parameters. In contrary, the generalized boosted ML, ensemble-based ML, and neural-net-specific DL models are more saturated accounting for both main and complex interaction effects between covariates. However, these models typically are overfitted with a smaller training data set, as is the case in our application, and thus require a larger set of tuning parameters to reduce the risk of generalization bias. Technical details for this step are provided as below.

## Model optimization

Model optimization step corresponds to Figure 3 Step: 4-5. This process involves experimenting with various combinations of hyperparameters for each model with respect to grid-search and cross-validation. For reproducibility, we assigned seed numbers to each model.

For RSF, we selected the hyperparameters for the number of variables to randomly sample as candidates at each split (i.e., a grid of values, each spaced apart by a distance of 2, ranging from the square root of the total number of features to the total number of features), the size of nodes (i.e., a grid of values, each spaced apart by a distance of 1, starting from 2 to 33), and depth of trees (i.e., a grid of values, each spaced apart by a distance of 500, ranging from 500 to 2500). Tuning parameters were selected via grid-search with respect to minimizing the out of bag (OOB) sample error. We used log-rank splitting with sampling with replacement bootstrap strategy for developing the forest. We kept the other parameters (e.g., node depth) that are pertinent to the survival data as default – we did not observe improvement in OOB error by changing these values from the defaults.

For RSB, we used the number of variables at each split equal to the number of features used in the model. The remaining tuning parameters’ domain remain as same as that of RSF. We used R-package **randomForestSRC** to implement both RSF and RSB.

For CISF, we used the univariate, quadratic test statistic for variable selection for splitting rather than selecting random. We observed superior numeric performances with the default parameters and thus kept them as default with respect to minimizing OOB error. We used R-package **partykit** to implement this method.

Cox-PH models were fitted with component wise likelihood-based boosting. In each boosting step the previous boosting steps are incorporated as an offset in penalized partial likelihood estimation, which is employed to obtain an update for one single parameter, i.e., one covariate, in every boosting step. Hyper parameters such as the number of boosting steps and penalty employed in each boosting step were selected by 10-fold CV with respect to maximizing the partial log likelihood. The model is implemented by R-package **CoxBoost.**

Cox-PH model with L1 norm penalty was fitted to obtain a parsimonious model. In the case of correlated predictors, as in our application, the model tends to pick one of the correlated covariates and discard the others shrinking them to exactly to zero. The LASSO penalty was selected via 10-fold CV minimizing the deviance of partial-likelihood for the Cox model. Similarly, Cox-PH model with L2 norm (Ridge) penalty was fitted where the penalty term is equivalent to square of the coefficients. Optimum ridge penalty was selected via 10-fold CV where the model shrinks the correlated predictor towards each other. Relaxed Cox-PH LASSO models were fitted with two tuning parameters – where the idea is to take a LASSO fitted object with respect to a value in the grid of sparse penalty, and then for each tuning parameter, refit the variables in the active set without any penalization. Relaxed model imposes another tuning parameter to obtain shrinkage between these two fitted objects to reduce the bias in the LASSO based coefficient estimates. Both hyper-parameters were selected via 10-fold CV. Cox-PH model with elastic net combines both Ridge and LASSO penalty improving the model fit by addressing multicollinearity more effectively. The corresponding tuning parameters were selected via 10-fold CV. Adaptive regularized Cox-PH models were fitted in two steps where at the first step, coefficient estimates based on a Cox-PH with L2-norm penalty were obtained and at the second step, another penalized Cox-PH model (e.g., Elastic-net) was fitted with parameter weights as reciprocal of the estimates of the first model fit. In both steps, tuning parameters were selected independently via 10-fold CV minimizing the deviance of partial likelihood. These models were implemented via R-package **glmnet.**

Adaptive Cox regression with minimax concave penalty were fitted in two steps where at the first stage parameter weights were estimated. At the second stage, a model with MCP penalty was fitted to obtain less biased regression coefficient estimates. In a similar spirit, smooth clipped absolute deviation penalty-based Cox regression was also fitted. In both cases, hyper parameters were selected via 10-fold CV. MNET penalty combines the contribution of MCP and L2 penalty; where the tuning parameters were selected via the combination of 10-fold CV (for MCP penalty) and grid search (for L2 penalty). Similarly, SNET penalty combines the contribution of SCAD and L2 penalty. In all regularized Cox regression models, cumulative baseline hazard functions were estimated via numerical approximation and linear predictors were estimated with respect to the corresponding model fit at optimum parameters. Predicted survival probabilities were obtained subsequently. These models were implemented via R-packages **glmnet** and **ncvreg.**

Deep learning-based survival models were fitted exploiting neural net architecture. Deep-Surv, Deep-Hit, and Deep-LogHaz models were fitted with rectified linear unit (ReLU) activation function with 2 or 3 hidden layers with a varied combination of nodes. The number of hidden nodes varies from one layer to another with the former layer having more nodes than the deeper layer in a descending order. This approach was chosen to minimize the computational burden. The unique combination of node sizes in each layer was chosen from a grid of values each spaced apart by 4, starting from 2 to 28. To prevent overfitting, a dropout layer was added where 10% individual nodes, chosen randomly, were excluded in the corresponding training runs. The models were fitted without any dropout layer too. Another hyper-parameter related to learning rates with values of 0.01, 0.02, and 0.05 were imposed to regulate the weights of neural network. To penalize large weights, the constant weight decay with values of 0.01 and 0.20, that to be multiplied with the original weights, were used. Batch size with 32 samples from the training dataset were used to estimate the error gradient before the model weights were updated. In addition, the number of epochs was set to 100 controlling the number of passes through the respective training dataset. All other tuning parameters were set as default. To reduce the computational burden, all the hyper-parameters were chosen by 3-fold CV minimizing the predicted survival risk. These models were implemented via R-packages **survivalmodels** which uses Python via another R-package **reticulate.**

A series of models were fitted for each model type. The model exhibiting the best performance (minimum error) with a certain configuration of hyperparameters was then considered as the optimal model for that specific type of model.

## Model selection by internal validation

Model selection step corresponds to Figure 3 Step: 6-8. The main objective was to select the best type of optimized ML model. We applied leave-one-out-cross-validation (LOOCV) for model evaluation. The idea of this internal validation was to train each model N (the number of subjects) times for each treatment (e.g., ven/aza) arm separately. Here each time, we left one subject out as a test set and used the remaining (N - 1) subjects’ information as the corresponding training sets. This resulted in N training sets. Note that we optimized hyperparameter tuning for each model, for each treatment arm, and for every training dataset, independently. We deployed parallel computation technique to minimize computational burden distributing the workloads over a Linux based machine with 38 cores and 380GB RAM using R-packages **Parallel** and **doParallel**. After optimizing each model, we refitted the model with the optimum set of hyperparameters and the prediction for overall survival/risk of death over 2-years for the respective test subject was obtained. All predicted results were concatenated row-wise resulting in a matrix of probabilities with a dimension of N by T (e.g., N refers to the total number of ven/aza subjects; T refers to the number of unique event times less than or equal to 2-years in the ven/aza analytical dataset). If needed, interpolation by last observation carried forward (LOCF) was used to enumerate survival probabilities for any user-specific intermediate time point. The evaluation metrics included dynamic area under the curve (AUC) of cumulative case dynamic control of receiver operative characteristics (ROC) (cAUC), incident case dynamic control ROC (iAUC), integrated Brier scores (IBS), time-dependent concordance (C) index and Brier score at one year survival. At the end of this step, the optimum model type was chosen comparing the numerical performances of each optimized model against the others.

## Model validation by independent test set

Adversarial validation step corresponds to Figure 3 Step: 10. The chosen model was retrained using the full analytical data set. To gauge the generalizability of the ML models, we applied the trained models (i.e., RSF for 7+3 and Cox-Boost for ven/aza) on unseen, independent validation sets of 7+3 (N = 14) and ven/aza (N = 30), respectively. These patients were treated in the UCHealth after we locked the analytical dataset and thus had not been used in any of our prior analyses. To check the extent of variation in data distribution between the validation and training sets, we ran the adversarial validation step. We fitted a logistic regression (logit link function) with a binary response having a value of 1 if data belongs to the validation set and 0 otherwise. We regressed this “pseudo” response on 114 features used in developing the ML models. Even though, the model was able to correctly predict the origin of dataset signaling an underlying potential drift, we applied the method to check the models’ performances in such a complex situation with non-homogeneous data. We reported the corresponding evaluation metrics.

## Patient-specific survival estimation

Survival estimation and uncertainty quantification step correspond to Figure 3 Step: 10-11. Once the best, optimized model was selected for each treatment arm, we refitted the corresponding model with the entire training dataset with appropriately reselecting the tuning parameters. For a given test patient, survival curve (in probability scale) over time was generated. Uncertainty in prediction was quantified via non-parametric bootstrap technique where we resampled the entire training dataset with replacement for 300 times and each time, we retrained the selected ML model optimizing the corresponding hyperparameters. We concatenated 300 survival curves stemming from each run for the test subject and 95% point-wise prediction interval was chosen with respect to 2.5^th^ and 97.5^th^ percentile values.

**SUPPLEMENTAL TABLE LEGENDS**

**Supplemental Table 1. Summary statistics for 7+3 and ven/aza treated patients** **at diagnosis**.

**Supplemental Table 2. Summary of clinical events (transfusions and ICU transfers) and toxicities occurring during the first 30 days of treatment for 7+3 and ven/aza treated patients.**

**Supplemental Table 3**. **Summary statistics for laboratory values at diagnosis (t_0_) and at the first AML response assessment between days 15 and 55 of treatment (t_15-55_).**

**Supplemental Table 4. Summary of phenotypic and genetic features recorded at diagnosis and day_15-55_ follow up.**

**Supplemental Table 5. Summary of the ELN AML response categories at the first t_15-55_ response assessment as well as a summary of the distribution of timing of these disease assessments.**

**Supplemental Table 6. Summary of lab features among AML patients in ven/aza training and external validation group.**

**Supplemental Table 7. Summary of phenotypic features among AML patients in ven/aza training and external validation group.**

**Supplementary Table 8. Summary of genetic features among AML patients in ven/aza training and external validation group.**

**Supplementary Table 9. Summary of post treatment events among AML patients in ven/aza training and external validation group.**

**Supplemental Table 10. Definition of ejection fraction (EF) toxicity grades.**

**Supplemental Table 11. Definition of genetic features.**

**Supplemental Table 12. List of features used in developing predictive models.**

**Supplemental Table 13. Features association with overall survival based on multivariate models.**

**Supplemental Table 1. Baseline summary demographics of 7+3 and ven/aza treated patients.**

|  | 7+3 | | ven/aza | *P*$\mathbf{value}_{\mathbf{TX}}^{\mathbf{c}}$ | $\mathbf{SMD}_{\mathbf{TX}}^{\mathbf{c}}$ |  |
| --- | --- | --- | --- | --- | --- | --- |
| N = | 111 | | 91 |  |  |  |
| ^b^Age, Median (IQR) [Range], y | **53.0 (18.0) [55.0]** | | **72.0 (12.0) [68.0]** | **<0.001** | 1.44 |  |
| ^b^Female, n (%) | 50 (45.0) | | 43 (47.3) | .86 | 0.04 |  |
| ^a^Non-Hispanic, n (%) | 99 (90.0) | | 82 (93.2) | .59 | 0.12 |  |
| Primary AML, n (%) | 90 (81.1) | | 62 (68.1) | .05 | 0.30 |  |
| Secondary AML (ICD-10 defined), n (%) | 21 (18.9) | | 29 (31.9) |  |  |  |
| General comorbidities |  | |  |  |  |  |
| ^b^Obesity, n (%) | | 13 (11.7) | 9 (9.9) | .85 | 0.06 |  |
| Coagulopathy, n (%) | | 16 (14.4) | 12 (13.2) | .96 | 0.04 |  |
| Chronic kidney disease, n (%) | | **3 (2.7)** | **14 (15.4)** | **<.01** | 0.45 |  |
| MDS, n (%) | **6 (5.4)** | | **15 (16.5)** | **.02** | 0.36 |  |
| Hypertension, n (%) | **29 (26.1)** | | **47 (51.6)** | **<.001** | 0.54 |  |
| Indication of T2D drug (Statin) utilization, n (%) | 9 (8.1) | | 9 (9.9) | .85 | 0.06 |  |
| ^a^ECOG Score, n (%) |  | |  |  |  |  |
| 0 | **15 (48.4)** | | **13 (22.0)** | **.02** | 0.57 |  |
| 1 | 15 (48.4) | | 31 (52.5) | .88 | 0.08 |  |
| 2 | **1 (3.2)** | | **15 (25.4)** | **.02** | 0.67 |  |
| ^a^ELN-2017 risk group, n (%) |  | |  |  |  |  |
| Adverse | **33 (34.4)** | | **53 (58.9)** | **<.001** | 0.51 |  |
| Intermediate | 21 (21.9) | | 16 (17.8) | .61 | 0.10 |  |
| Favorable | **42 (37.8)** | | **21 (23.1)** | **.01** | 0.33 |  |

**^a^**Summary statistics (i.e., median, IQR, range, proportion (%), *P* value, standardized mean difference (SMD)) were calculated after excluding missing cases.

**^b^**Features used in ML models.

**^c^**Superscript “TX” stands denoting differences of the corresponding variable between 7+3 and ven/aza

**Remarks:**

- **t_0_** indicates treatment (TX) start date
- The term “v/a” was used interchangeably with ven/aza for brevity and notational convenience.
- Missing variables at **t_0_**: ECOG: 80 7+3 and 32 v/a; ELN risk: 15 7+3 and 1 v/a;
- Mann-Whitney U-test and Fisher’s exact test if counts are less than five or Chi-Squared tests were used for numerical and categorical variables respectively. Standardized mean differences (SMD) by treatments were reported.
- *P* values were not adjusted for multiplicity.

**Supplemental Table 2. Summary table for organ status and hospital events.**

|  | Time | | 7+3 | | ven/aza | | *P* $\mathbf{value}_{\mathbf{TX}}^{\mathbf{c}}$ | | $\mathbf{SMD}_{\mathbf{TX}}^{\mathbf{c}}$ | |
| --- | --- | --- | --- | --- | --- | --- | --- | --- | --- | --- |
| N = |  | 111 | | 91 | |  | |  | |  |
| Transfusions |  |  | |  | |  | |  | |  |
| ^b^Platelet bags, Median (IQR) [Range], Count | **t_ind_** | **9.0 (8.5) [66.0]** | | **1.0 (5.0) [62]** | | **<.001** | | **0.80** | |  |
| ^b^Red blood cell bags, Median (IQR) [Range], Count | **t_ind_** | **8.0 (4.0) [25.0]** | | **3.0 (3.5) [20.0]** | | **<.001** | | **1.06** | |  |
| ^b^Length of stay, Median (IQR) [Range], Days | **t_ind_** | **30.0 (8.0)[105.0]** | | **9.0 (5.0) [26.0]** | | **<.001** | | **2.39** | |  |
| ^b^ICU transfer during induction, n (%) | **t_ind_** | **17 (15.3)** | | **5 (5.5)** | | **.04** | | **0.33** | |  |
| Toxicity (CTCAE), n (%) |  |  | |  | |  | |  | |  |
| ^ba^ALT ≥Grade 1 | **t_<30_** | **62 (55.9)** | | **16 (17.8)** | | **<.001** | | **0.86** | |  |
| ^b^AST ≥Grade 2 | **t_<30_** | **14 (12.6)** | | **4 (4.4)** | | **.05** | | **0.30** | |  |
| ^b^Bilirubin ≥Grade 2 | **t_<30_** | 26 (23.4) | | 15 (16.5) | | .30 | | 0.17 | |  |
| ^b^Anemia ≥Grade 3 | **t_<30_** | **110 (99.1)** | | **82 (90.1)** | | **<.01** | | **0.41** | |  |
| ^ba^Chronic kidney disease ≥Grade 3 | **t_<30_** | 5 (27.8) | | 6 (19.4) | | .50 | | 0.20 | |  |
| ^b^Creatinine ≥Grade 2 | **t_<30_** | 9 (8.1) | | 10 (11.0) | | .65 | | 0.10 | |  |
| ^ba^Ejection fraction ≥Grade 1 | **t_<30_** | 13 (31.0) | | 3 (16.7) | | .35 | | 0.34 | |  |
| ^b^Febrile neutropenia ≥Grade 3 | **t_<30_** | **86 (77.5)** | | **20 (22.0)** | | **<.001** | | **1.33** | |  |
| ^b^Neutrophils ≥Grade 4 | **t_<30_** | 109 (98.2) | | 88 (96.7) | | .66 | | 0.10 | |  |
| ^ba^Proteinuria ≥Grade 2 | **t_<30_** | 19 (18.4) | | 13 (21.3) | | .81 | | 0.07 | |  |
| ^b^Thrombocytopenia ≥Grade 4 | **t_<30_** | **110 (99.1)** | | **61 (67.0)** | | **<.001** | | **0.95** | |  |
| Readmission or occurrences after discharge, n (%) |  |  | |  | |  | |  | |  |
| ^b^Hospital readmission by 30d FUP Bx date | **t_<30_** | **2 (1.8)** | | **26 (28.6)** | | **<.001** | | **0.80** | |  |
| ^b^ICU readmission by 30d FUP Bx date | **t_<30_** | 1 (0.9) | | 2 (2.2) | | .59 | | 0.11 | |  |
| ^b^At least one bag of RBC transfusion by 30d FUP Bx date | **t_<30_** | **1 (0.9)** | | **20 (22.0)** | | **<.001** | | **0.70** | |  |
| ^†^At least one bag of platelet transfusion by 30d FUP Bx date | **t_<30_** | **1 (0.9)** | | **16 (17.6)** | | **<.001** | | **0.60** | |  |

**^a^**Summary statistics (i.e., median, IQR, range, *P* value, standardized mean difference (SMD)) were calculated after excluding missing data elements.

**^b^**Features used in ML models.

**^c^**Superscript “TX” stands denoting differences of the corresponding variable between 7+3 and ven/aza

**Remarks:**

- **t_15-55,_ t_<30_, and t_ind_** indicate FUP Bx, within 1-30 days post-TX, and induction period respectively.
- **Missing toxicity within the first 30 days post initiating treatment t_<30_**: ALT: 1 v/a; CKD: 93 7+3 and 60 v/a; Ejection-fraction: 69 7+3 and 73 v/a; Proteinuria: 8 7+3 and 30 v/a. **Supplemental Table 2.**
- *P* values were not adjusted for multiplicity.

**Supplemental Table 3. Summary of patient features at diagnosis and day_15-55_ follow up.**

|  | Time | 7+3 | ven/aza | *P*- $\mathbf{value}_{\mathbf{TX}}^{\mathbf{c}}$ | $\mathbf{SMD}_{\mathbf{TX}}^{\mathbf{c}}$ | *P* value^0-30^  [7+3]  [ven/aza] |
| --- | --- | --- | --- | --- | --- | --- |
| N = |  | 111 | 91 |  |  |  |
| Labs, Median (IQR) [Range] |  |  |  |  |  |  |
| Alanine aminotransferase, U/L | **t_0_** | **22.0 (19.5)**  **[327.0]** | **16.0 (10.0)**  **[54.0]** | **<.01** | **0.42** |  |
|  | **t_15-55_** | **30.0 (25.0)**  **[368.0]** | **12.0 (5.8)**  **[144.0]** | **<.001** | **0.61** | [<.01] [<.001] |
| ^b^Aspartate aminotransferase, U/L | **t_0_** | 22.0 (18.5)  [176.0] | 20.0 (16.0)  [77.0] | .29 | 0.22 |  |
|  | **t_15-55_** | **20.0 (16.5)**  **[315.0]** | **15.0 (7.0)**  **[111.0]** | **<.001** | **0.40** | [.15]  [<.001] |
| ^b^Albumin, g/dL | **t_0_** | 3.6 (0.7)  [2.6] | 3.6 (0.8)  [2.3] | .60 | 0.09 |  |
|  | **t_15-55_** | **3.5 (0.9)**  **[3.0]** | **3.7 (0.7)**  **[3.0]** | **.01** | **0.40** | [<.001]  [.12] |
| ^b^Bilirubin, mg/dL | **t_0_** | 0.6 (0.5)  [2.9] | 0.6 (0.4)  [3.5] | .82 | 0.07 |  |
|  | **t_15-55_** | **0.6 (0.4)**  **[2.8]** | **0.7 (0.4)**  **[1.9]** | **<.001** | **0.35** | [.10]  [<.01] |
| ^b^Calcium, mg/dL | **t_0_** | 9.0 (0.8)  [2.7] | 9.0 (0.7)  [5.1] | .47 | 0.20 |  |
|  | **t_15-55_** | 9.0 (0.8)  [3.2] | 9.0 (0.6)  [2.7] | .65 | 0.16 | [.35]  [.26] |
| ^b^Creatinine, mg/dL | **t_0_** | 0.9 (0.3)  [2.4] | 1.0 (0.5)  [3.4] | .07 | 0.21 |  |
|  | **t_15-55_** | **0.7 (0.3)**  **[2.5]** | **0.8 (0.4)**  **[1.7]** | **<.01** | **0.19** | [<.001] [<.001] |
| ^ba^Fibrinogen, mg/dL | **t_0_** | 405.0 (189.0)  [663.0] | 373.0 (173.5)  [734.0] | .30 | 0.12 |  |
|  | **t_15-55_** | **390.5 (174.0)**  **[717.0]** | **499.5 (304.8)**  **[478.0]** | **<.01** | **0.71** | [.37]  [.03] |
| ^b^Hemoglobin, g/dL | **t_0_** | **9.3 (2.5)**  **[12.7]** | **8.5 (2.8)**  **[9.5]** | **.04** | **0.29** |  |
|  | **t_15-55_** | **9.7 (2.2)**  **[6.7]** | **9.0 (2.2)**  **[8.2]** | **.01** | **0.33** | [.48]  [.21] |
| ^b^White blood cell, 10^9^/L | **t_0_** | **13.9 (55.7)**  **[681.4]** | **3.9 (28.5)**  **[255.4]** | **.01** | **0.30** |  |
|  | **t_15-55_** | **4.0 (5.6)**  **[26.3]** | **0.9 (1.0)**  **[7.1]** | **<.001** | **1.12** | [<.001] [<.001] |
| ^ba^Lymphocytes,10^9^/L | **t_0_** | **2.9 (5.7)**  **[55.5]** | **1.4 (2.3)**  **[37.2]** | **<.01** | **0.29** |  |
|  | **t_15-55_** | **1.0 (0.6)**  **[4.1]** | **0.6 (0.4)**  **[3.2]** | **<.001** | **0.72** | [<.001] [<.001] |
| ^ba^Neutrophils (ANC), 10^9^/L | **t_0_** | **1.5 (4.9)**  **[34.3]** | **0.8 (2.1)**  **[28.0]** | **.02** | **0.34** |  |
|  | **t_15-55_** | **1.9 (3.9)**  **[23.0]** | **0.1 (0.5)**  **[4.1]** | **<.001** | **1.00** | [.87]  [<.001] |

**Supplemental Table 3, cont’d**

|  | Time | 7+3 | ven/aza | *P*- $\mathbf{value}_{\mathbf{TX}}^{\mathbf{c}}$ | $\mathbf{SMD}_{\mathbf{TX}}^{\mathbf{c}}$ | *P* value^0-30^  [7+3]  [ven/aza] |
| --- | --- | --- | --- | --- | --- | --- |
| N = |  | 111 | 91 |  |  |  |
| Labs, Median (IQR) [Range] |  |  |  |  |  |  |
| ^b^Blast, % | **t_0_** | 59.5 (43.3)  [94.0] | 56.0 (42.0)  [92.5] | .55 | 0.07 |  |
|  | **t_15-55_** | 2.0 (2.0)  [94.5] | 1.5 (3.5)  [62.5] | .69 | 0.07 | [<.001]  [<.001] |
| ^b^Platelets, 10^9^/L | **t_0_** | 46.0 (35.5)  [404.0] | 53.0 (60.5)  [241.0] | .77 | 0.02 |  |
|  | **t_15-55_** | **252.0 (359.5)**  **[1400.0]** | **154.0 (234.5)**  **[907.0]** | **<.001** | **0.54** | [<.001]  [<.001] |
| ^ba^Lactate dehydrogenase, U/L | **t_0_** | 344.0 (406.5)  [3741.0] | 279.0 (262.0)  [2208.0] | .09 | 0.15 |  |
|  | **t_15-55_** | **207.0 (89.5)**  **[1094.0]** | **173.5 (51.5)**  **[1923.0]** | **.02** | **0.01** | [<.001]  [<.001] |
| ^b^Potassium, mmol/L | **t_0_** | 3.7 (0.5)  [3.7] | 3.8 (0.6)  [2.9] | .12 | 0.14 |  |
|  | **t_15-55_** | 3.9 (0.4)  [1.8] | 4.0 (0.3)  [2.1] | .16 | 0.22 | [<.001]  [<.001] |
| ^ba^Uric-acid, mg/dL | **t_0_** | 5.1 (3.0)  [19.7] | 4.7 (2.3)  [14.4] | .16 | 0.17 |  |
|  | **t_15-55_** | **4.5 (2.4)**  **[6.9]** | **3.5 (1.3)**  **[5.1]** | **<.001** | **0.68** | [<.001]  [<.001] |

**^a^**Summary statistics (i.e., median, IQR, range, *P* value, standardized mean difference (SMD)) were calculated after excluding missing data elements.

**^b^**Features used in machine-learning (ML) models.

**^c^**Superscript “TX” stands denoting differences of the corresponding variable between 7+3 and ven/aza

**Remarks:**

- **t_0_** and **t_15-55_** indicates TX start date and FUP Bx respectively.
- The term “v/a” was used interchangeably with ven/aza for brevity and notational convenience.
- Missing labs at FUP Bx date t_15-55_:: Phosphorus: 2 7+3; Fibrinogen: 55 7+3 and 67 v/a; ANC: 2 7+3 and 5 v/a; Uric-acid: 18 7+3 and 13 v/a; LDH: 15 7+3 and 13 v/a; Lymphocytes: 3 7+3 and 4 v/a.
- Missing labs (i.e., Phosphorus, ANC, Uric-acid, LDH, and lymphocytes) were imputed by median values of the respective cohort in ML models.
- Mann-Whitney U-test was used. Standardized mean differences (SMD) by treatments were reported.
- Paired Wilcoxon signed rank test was used for detecting differences between **t_0_** and **t_15-55._**
- *P* values were not adjusted for multiplicity.

**Supplemental Table 4. Summary of phenotypic and genetic features recorded at diagnosis and day_15-55_ follow up.**

|  | Time | 7+3 | ven/aza | *P*$\mathbf{value}_{\mathbf{TX}}^{\mathbf{c}}$ | $\mathbf{SMD}_{\mathbf{TX}}^{\mathbf{c}}$ | *P* value^0-30^  [7+3][ ven/aza] |
| --- | --- | --- | --- | --- | --- | --- |
| N = |  | 111 | 91 |  |  |  |
| AML Flow cytometry, n (%) |  |  |  |  |  |  |
| ^b^CD123 | **t_0_** | **13 (11.7)** | **36 (39.6)** | **<.001** | **0.67** |  |
|  | **t_15-55_** | **8 (7.2)** | **17 (18.7)** | **.03** | **0.35** | **[.33] [<.01]** |
| ^b^CD117 | **t_0_** | **76 (68.5)** | **77 (84.6)** | **.01** | **0.39** |  |
|  | **t_15-55_** | 18 (16.2) | 24 (26.4) | .11 | 0.25 | [<.001] [<.001] |
| ^b^CD11B | **t_0_** | 25 (22.5) | 27 (29.7) | .32 | 0.16 |  |
|  | **t_15-55_** | 8 (7.2) | 10 (11.0) | .49 | 0.13 | [<.01] [<.01] |
| ^b^CD33 | **t_0_** | **51 (45.9)** | **69 (75.8)** | **<.001** | **0.64** |  |
|  | **t_15-55_** | 17 (15.3) | 24 (26.4) | .08 | 0.28 | [<.001] [<.001] |
| ^b^CD34 | **t_0_** | 60 (54.1) | 61 (67.0) | .08 | 0.27 |  |
|  | **t_15-55_** | 19 (17.1) | 22 (24.2) | .29 | 0.18 | [<.001] [<.001] |
| CD36 | **t_0_** | **2 (1.8)** | **8 (8.8)** | **.05** | **0.32** |  |
|  | **t_15-55_** | **0 (0.0)** | **4 (4.4)** | **.04** | **0.30** | [.48] [.39] |
| ^b^CD38 | **t_0_** | **60 (54.1)** | **72 (79.1)** | **<.001** | **0.55** |  |
|  | **t_15-55_** | 17 (15.3) | 25 (27.5) | .05 | 0.30 | [<.001] [<.001] |
| ^b^CD13 | **t_0_** | **61 (55.0)** | **72 (79.1)** | **<.001** | **0.53** |  |
|  | **t_15-55_** | 20 (18.0) | 27 (29.7) | .08 | 0.28 | [<.001] [<.001] |
| ^b^CD14 | **t_0_** | 7 (6.3) | 5 (5.5) | 1.00 | 0.03 |  |
|  | **t_15-55_** | 2 (1.8) | 2 (2.2) | 1.00 | 0.03 | [.18] [.37] |
| ^b^CD15 | **t_0_** | 12 (10.8) | 7 (7.7) | .61 | 0.11 |  |
|  | **t_15-55_** | 4 (3.6) | 1 (1.1) | .38 | 0.17 | [.08] [.08] |
| ^b^CD45 | **t_0_** | 67 (60.4) | 48 (52.7) | .35 | 0.15 |  |
|  | **t_15-55_** | 12 (10.8) | 7 (7.7) | .61 | 0.11 | [<.001] [<.001] |
| ^b^CD56 | **t_0_** | 12 (10.8) | 18 (19.8) | .11 | 0.25 |  |
|  | **t_15-55_** | 6 (5.4) | 3 (3.3) | .52 | 0.10 | [.15] [<.01] |
| ^b^CD64 | **t_0_** | 25 (22.5) | 28 (30.8) | .24 | 0.19 |  |
|  | **t_15-55_** | 5 (4.5) | 8 (8.8) | .26 | 0.17 | [<.001] [<.001] |
| ^b^CD7 | **t_0_** | 14 (12.6) | 19 (20.9) | .17 | 0.22 |  |
|  | **t_15-55_** | 3 (2.7) | 5 (5.5) | .47 | 0.14 | [.01] [<.01] |
| ^b^MPO | **t_0_** | 46 (41.4) | 39 (42.9) | .95 | 0.03 |  |
|  | **t_15-55_** | 4 (3.6) | 1 (1.1) | .38 | 0.17 | [<.001] [<.001] |
| ^b^HLADR | **t_0_** | **56 (50.5)** | **65 (71.4)** | **<.01** | **0.44** |  |
|  | **t_15-55_** | 18 (16.2) | 21 (23.1) | 0.29 | 0.17 | [<.001] [<.001] |
| ^b^Cytogenetic risk group, n (%) |  |  |  |  |  |  |
| Good | **t_0_** | **19 (17.1)** | **3 (3.3)** | **<.01** | **0.47** |  |
|  | **t_15-55_** | 1 (0.9) | 0 (0.0) | 1.00 | 0.14 | [<.001] [.25] |
| Intermediate | **t_0_** | 56 (50.5) | 42 (46.2) | .64 | 0.09 |  |
|  | **t_15-55_** | **86 (77.5)** | **56 (61.5)** | **.02** | **0.35** | [<.001] [.02] |
| Poor | **t_0_** | 20 (18.0) | 25 (27.5) | .15 | 0.23 |  |
|  | **t_15-55_** | 7 (6.3) | 11 (12.1) | .24 | 0.20 | [<.01] [<.01] |
| Indeterminant | **t_0_** | 23 (20.7) | 24 (26.4) | .44 | 0.13 |  |
|  | **t_15-55_** | 17 (15.3) | 25 (27.5) | .05 | 0.30 | [.36] [1.00] |

**Supplemental Table 4, cont’d**

**^a^**Summary statistics (i.e., proportion (%), *P* value, SMD) were calculated after excluding missing cases.

**^b^**Features used in ML models.

**^c^**Superscript “TX” stands denoting differences of the corresponding variable between 7+3 and ven/aza.

**^d^**Features at **t_15-55_** are missing.

**^e^***P* values for paired data were calculated based on complete cases across **t_0_** and **t_15-55_**. Tests were not performed (abbreviated by [np]) for variables with too few samples (less than 4) and cases with zero-variation.

**Remarks:**

- **t_0_** and **t_15-55_** indicates TX start date and FUP Bx respectively.
- The term “v/a” was used interchangeably with ven/aza for brevity and notational convenience.
- Missing diagnostic markers at **t_0_** :: ELN risk: 15 7+3 and 1 v/a; ECOG: 80 7+3 and 32 v/a; FLT3: 18 7+3 and 13 v/a; RUNX: 30 7+3 and 10 v/a; CBFB: 8 7+3 and 10 v/a; NPM1: 20 7+3 and 13 v/a; IDH1: 22 7+3 and 13 v/a; IDH2: 23 7+3 and 13 v/a; PML: 31 7+3 and 19 v/a; TP53: 39 7+3 and 11 v/a; EGR1: 13 7+3 and 11 v/a; MLL: 13 7+3 and 15 v/a; 8centromere: 19 7+3 and 16 v/a; 7centromere: 9 7+3 and 11 v/a.

|  | Time | 7+3 | ven/aza | *P*- $\mathbf{value}_{\mathbf{TX}}^{\mathbf{c}}$ | $\mathbf{SMD}_{\mathbf{TX}}^{\mathbf{c}}$ | *P* value^0-30^  [7+3][ven/aza] |
| --- | --- | --- | --- | --- | --- | --- |
| Complex cytogenetics, n (%) | **t_0_** | 19 (17.1) | 22 (24.2) | .29 | 0.18 |  |
|  | **t_15-55_** | 7 (6.3) | 8 (8.8) | .69 | 0.09 | [<.01] [<.01] |
| Monosomal karyotype, n (%) | **t_0_** | 11 (9.9) | 12 (13.2) | .61 | 0.10 |  |
|  | **t_15-55_** | 3 (2.7) | 5 (5.5) | .47 | 0.14 | [.01] [.05] |
| ^ba^FLT3, n (%) | **t_0_** | 32 (34.4) | 17 (27.8) | .10 | 0.28 |  |
|  | **t_15-55_** | **2 (11.1)** | **3 (75.0)** | **.02** | **1.69** | [<.001]^e^ [np] |
| ^ba^RUNX, n (%) | **t_0_** | 29 (35.8) | 26 (32.1) | .74 | 0.08 |  |
|  | **t_15-55_** | 1 (50.0) | 2 (100.0) | 1.00 | 1.41 | [np] [np] |
| ^ba^CBFB, n (%) | **t_0_** | 24 (23.3) | 9 (11.1) | .05 | 0.33 |  |
|  | **t_15-55_** | 5 (20.0) | 2 (33.3) | .60 | 0.31 | [<.001]^e^[<.001]^e^ |
| ^ba^NPM1, n (%) | **t_0_** | 33 (36.3) | 25 (32.1) | .68 | 0.09 |  |
|  | **t_15-55_** | **1 (5.6)** | **2 (66.7)** | **.04** | **1.65** | [<.001] ^e^ [np] |
| ^ad^IDH1, n (%) | **t_0_** | 5 (5.6) | 10 (12.8) | .17 | 0.25 |  |
| ^ba^IDH2, n (%) | **t_0_** | 12 (13.6) | 14 (17.9) | .58 | 0.12 |  |
|  | **t_15-55_** | 1 (16.7) | 1 (25.0) | 1.00 | 0.21 | [.25]^e^ [1.00]^e^ |
| ^ad^PML, n (%) | **t_0_** | **0 (0.0)** | **4 (5.6)** | **.05** | **0.34** |  |
| ^a^TP53, n (%) | **t_0_** | **2 (2.8)** | **13 (16.2)** | **<.01** | **0.47** |  |
|  | **t_15-55_** | 0 (0.0) | 1 (14.3) | 1.00 | 0.58 | [np] [.13]^e^ |
| Fluorescence in situ hybridization (FISH), n (%) |  |  |  |  |  |  |
| ^ba^EGR1 | **t_0_** | **5 (5.1)** | **13 (16.2)** | **.02** | **0.37** |  |
|  | **t_15-55_** | 3 (50.0) | 6 (42.9) | 1.00 | 0.14 | [np] [.07]^e^ |
| ^ba^MLL | **t_0_** | 14 (14.3) | 15 (19.7) | .45 | 0.15 |  |
|  | **t_15-55_** | 4 (28.6) | 8 (66.7) | .11 | 0.83 | [.02] ^e^ [.02]^e^ |
| ^ba^8Centromere | **t_0_** | 16 (17.4) | 20 (26.7) | .21 | 0.23 |  |
|  | **t_15-55_** | 6 (35.3) | 12 (54.5) | .38 | 0.39 | [.01] ^e^ [.05]^e^ |
| ^a^7Centromere | **t_0_** | 9 (8.8) | 16 (20.0) | .05 | 0.32 |  |
|  | **t_15-55_** | 5 (50.0) | 12 (63.2) | .69 | 0.27 | [.25] ^e^ [<.01]^e^ |

**Supplemental Table 4, cont’d**

**Remarks (cont’d):**

- Missing biomarkers at FUP Bx date **t_15-55_:: FL**T3: 93 7+3 and 87 v/a; RUNX 109 7+3 and 89 v/a; CBFB: 86 7+3 and 85 v/a; NPM1: 93 7+3 and 88 v/a; IDH2: 105 7+3 and 87 v/a; TP53: 110 7+3 and 84 v/a; EGR1: 105 7+3 and 77 v/a; MLL: 97 7+3 and 79 v/a; 8centromere: 94 7+3 and 69 v/a; 7centromere: 101 7+3 and 72 v/a; IDH1 and PML were missing.
- Missing classes were used as a separate category in ML models.
- Fisher’s exact test if counts are less than five or Chi-Squared tests was used for categorical variables. Standardized mean differences (SMD) by treatments were reported.
- McNemar test for paired samples was used for detecting differences between **t_0_** and **t_15-55._**
- *P* values were not adjusted for multiplicity.

**Supplemental Table 5. Summary of the AML response categories at the first t_15-55_ response assessment as well as a summary of the distribution of timing of these disease assessments.**

|  | Time | 7+3 | ven/aza | *P* value_TX_ | SMD_TX_ |
| --- | --- | --- | --- | --- | --- |
| ^b^Follow-up biopsy 30d responses, n (%) |  |  |  |  |  |
| Complete remission | **t_15-55_** | **61 (55.0)** | **9 (9.9)** | <.001 | 1.10 |
| Complete remission with incomplete hematologic recovery | **t_15-55_** | **28 (25.2)** | **39 (42.9)** | .01 | 0.38 |
| Morphological leukemia-free state | **t_15-55_** | **5 (4.5)** | **22 (24.2)** | <.001 | 0.59 |
| Progressive disease | **t_15-55_** | 2 (1.8) | 0 (0.0) | .50 | 0.19 |
| Stable disease | **t_15-55_** | 15 (13.5) | 21 (23.1) | .11 | 0.25 |
| ^b^Date differences between TX start date and 30d FUP Bx date, n (%) | **t_15-55_** |  |  |  |  |
| [15d,25d] | **t_15-55_** | 20 (18.0) | 14 (15.4) | .76 | 0.07 |
| (25d,30d] | **t_15-55_** | **36 (32.4)** | **71 (78.0)** | <.001 | 1.03 |
| (30d,55d] | **t_15-55_** | **55 (49.5)** | **6 (6.6)** | <.001 | 1.09 |

^b^Features used in ML models.

**Remarks:**

- **t_15-55,_ t_<30_,** and **t_ind_** indicate FUP Bx, within 1-30 days post-TX, and induction period respectively.
- The term “v/a” was used interchangeably with ven/aza for brevity and notational convenience.
- Mann-Whitney U-test and Fisher’s exact test if counts are less than five or Chi-Squared tests were used for numerical and categorical variables respectively. Standardized mean differences (SMD) by treatments were reported.
- *P* values were not adjusted for multiplicity.

**Supplemental Table 6. Summary of lab features among AML patients in ven/aza training and external validation group.**

|  | Time | Training | Validation | SMD |
| --- | --- | --- | --- | --- |
| N = |  | 91 | 30 |  |
| Demographics |  |  |  |  |
| ^b^Age, Mean (SD), y | t_0_ | 69.5 (14.2) | 69.3 (8.8) | 0.02 |
| ^b^Male, n (%) | t_0_ | 48 (52.7) | 17 (56.7) | 0.08 |
| Labs, Mean (SD) |  |  |  |  |
| ^b^Albumin, g/dL | t_0_ | 3.7 (0.5) | 3.7 (0.6) | 0.06 |
|  | t_15-55_ | 3.6 (0.6) | 3.7 (0.5) | 0.09 |
| ^b^Aspartate aminotransferase (AST), U/L | t_0_ | 25.0 (14.8) | 26.9 (18.1) | 0.11 |
|  | t_15-55_ | 18.1 (14.3) | 16.5 (6.2) | 0.14 |
| ^b^Bilirubin, mg/dL | t_0_ | 0.7 (0.5) | 0.8 (0.6) | 0.12 |
|  | t_15-55_ | 0.8 (0.4) | 0.9 (0.5) | 0.31 |
| ^b^Blast, % | t_0_ | 52.7 (24.4) | 42.4 (27.0) | 0.40 |
|  | t_15-55_ | 7.1 (13.7) | 5.1 (8.9) | 0.18 |
| ^b^Calcium, mg/dL | t_0_ | 9.0 (0.7) | 8.8 (0.7) | 0.29 |
|  | t_15-55_ | 9.0 (0.5) | 8.9 (0.6) | 0.22 |
| ^b^Creatinine, mg/dL | t_0_ | 1.1 (0.5) | 0.9 (0.3) | 0.32 |
|  | t_15-55_ | 0.9 (0.3) | 0.9 (0.3) | 0.04 |
| ^ba^Fibrinogen, mg/dL | t_0_ | 397.3. (150.6) | 386.7 (137.5) | 0.07 |
| ^b^Hemoglobin, g/dL | t_0_ | 9.0 (1.9) | 9.0 (1.7) | 0.02 |
|  | t_15-55_ | 9.2 (1.5) | 8.8 (1.6) [8.2] | 0.23 |
| ^ba^Lactate dehydrogenase, U/L | t_0_ | 422.9 (405.7) | 389.6 (567.7) | 0.07 |
|  | t_15-55_ | 227.2 (224.0) | 194.5 (89.3) | 0.19 |
| ^ba^Lymphocytes, 10^9^/L | t_0_ | 3.3 (4.9) | 1.8 (2.2) | 0.39 |
|  | t_15-55_ | 0.7 (0.4) | 0.8 (0.6) | 0.17 |
| ^ba^Neutrophils (ANC), 10^9^/L | t_0_ | 2.3 (4.3) | 1.0 (1.2) | 0.41 |
|  | t_15-55_ | 0.4 (0.7) | 0.4 (0.5) | 0.06 |
| ^ba^Phosphorus, mg/dL | t_0_ | 3.3 (0.7) | 3.2 (0.7) | 0.04 |
|  | t_15-55_ | 3.6 (0.7) | 3.3 (0.6) | 0.43 |
| ^b^Platelets, 10^9^/L | t_0_ | 67.7 (53.8) | 70.9 (62.4) | 0.05 |
|  | t_15-55_ | 186.1 (164.2) | 138.2 (127.2) | 0.33 |
| ^b^Potassium, mmol/L | t_0_ | 3.8 (0.5) | 3.8 (0.3) | 0.05 |
|  | t_15-55_ | 4.0 (0.3) | 3.8 (0.4) | 0.51 |
| ^ba^Uric-acid, mg/dL | t_0_ | 5.3 (2.6) | 3.9 (1.4) | 0.66 |
|  | t_15-55_ | 3.6 (1.0) | 3.5 (1.0) | 0.06 |
| ^b^White blood cell, 10^9^/L | t_0_ | 27.7 (49.3) | 7.4 (14.5) | 0.56 |
|  | t_15-55_ | 1.2 (1.3) | 1.1 (1.0) | 0.05 |

**^a^**Summary statistics (i.e., median, IQR, range, *P* value, standardized mean difference (SMD)) were calculated after excluding missing data elements.

**^b^**Features used in ML models.

**Supplemental Table 7. Summary of phenotypic features among AML patients in ven/aza training and external validation group.**

|  | Time | Training | Validation | SMD |
| --- | --- | --- | --- | --- |
| N = |  | 91 | 30 |  |
| AML Flow cytometry, n (%) |  |  |  |  |
| ^b^CD123 | t_0_ | 36 (39.6) | 10 (33.3) | 0.13 |
|  | t_15-55_ | 17 (18.7) | 15 (50.0) | 0.70 |
| ^b^CD117 | t_0_ | 77 (84.6) | 27 (90.0) | 0.16 |
|  | t_15-55_ | 24 (26.4) | 15 (50.0) | 0.70 |
| ^b^CD11B | t_0_ | 27 (29.7) | 6 (20.0) | 0.23 |
|  | t_15-55_ | 10 (11.0) | 9 (30.0) | 0.49 |
| ^b^CD33 | t_0_ | 69 (75.8) | 23.0 (76.7) | 0.02 |
|  | t_15-55_ | 24 (26.4) | 17 (56.7) | 0.65 |
| ^b^CD34 | t_0_ | 61 (67.0) | 23 (76.7) | 0.02 |
|  | t_15-55_ | 22 (24.2) | 13 (43.3) | 0.41 |
| ^b^CD38 | t_0_ | 72 (79.1) | 26 (86.7) | 0.20 |
|  | t_15-55_ | 25 (27.5) | 16 (53.3) | 0.55 |
| ^b^CD13 | t_0_ | 72 (79.1) | 25 (83.3) | 0.11 |
|  | t_15-55_ | 27 (29.7) | 16 (53.3) | 0.50 |
| ^b^CD14 | t_0_ | 5 (5.5) | 1 (3.3) | 0.11 |
|  | t_15-55_ | 2 (2.2) | 2 (6.7) | 0.22 |
| ^b^CD15 | t_0_ | 7 (7.7) | 3 (10.0) | 0.08 |
|  | t_15-55_ | 1 (1.1) | 1 (3.3) | 0.15 |
| ^b^CD45 | t_0_ | 48 (52.7) | 13 (43.3) | 0.19 |
|  | t_15-55_ | 7 (7.7) | 3 (10.0) | 0.08 |
| ^b^CD56 | t_0_ | 18 (19.8) | 8 (26.7) | 0.16 |
|  | t_15-55_ | 3 (3.3) | 8 (26.7) | 0.69 |
| ^b^CD64 | t_0_ | 28 (30.8) | 5 (16.7) | 0.34 |
|  | t_15-55_ | 8 (8.8) | 6 (20.0) | 0.32 |
| ^b^CD7 | t_0_ | 19 (20.9) | 7 (23.3) | 0.06 |
|  | t_15-55_ | 5 (5.5) | 4 (13.3) | 0.27 |
| ^b^MPO | t_0_ | 39 (42.9) | 13 (43.3) | 0.01 |
|  | t_15-55_ | 1 (1.1) | 1 (3.3) | 0.15 |
| ^b^HLADR | t_0_ | 65 (71.4) | 21 (70.0) | 0.03 |
|  | t_15-55_ | 21 (23.1) | 15 (50.0) | 0.58 |

**^b^**Features used in ML models.

**Supplementary Table 8. Summary of genetic features among AML patients in ven/aza training and external validation group.**

|  | Time | Training | Validation | SMD |
| --- | --- | --- | --- | --- |
| N = |  | 91 | 30 |  |
| ^ba^Cytogenetic risk group, n (%) |  |  |  |  |
| Good | t_0_ | 3 (3.3) | 0 (0.0) | 0.26 |
|  | t_15-55_ | 0 (0.0) | 0 (0.0) | <0.01 |
| Intermediate | t_0_ | 42 (46.2) | 10 (33.3) | 0.26 |
|  | t_15-55_ | 56 (61.5) | 15 (50.0) | 0.23 |
| Poor | t_0_ | 25 (27.5) | 12 (40.0) | 0.27 |
|  | t_15-55_ | 11 (12.1) | 6 (20.0) | 0.22 |
| Indeterminant | t_0_ | 24 (26.4) | 10 (33.3) | 0.15 |
|  | t_15-55_ | 25 (27.5) | 0 (0.0) | 0.87 |
| ^ba^FLT3, n (%) |  |  |  |  |
| Mutation (+ve) | t_0_ | 17 (18.7) | 3 (10.0) | 2.57 |
| Wild-type (-ve) |  | 61 (67.0) | 0 (0.0) |  |
| Mutation (+ve) | t_15-55_ | 3 (3.3) | 2 (6.7) | 0.22 |
| Wild-type (-ve) |  | 1 (1.1) | 1 (3.3) |  |
| ^ba^RUNX, n (%) |  |  |  |  |
| Mutation (+ve) | t_0_ | 26 (28.6) | 10 (33.3) | 0.23 |
| Wild-type (-ve) |  | 55 (60.4) | 15 (50.0) |  |
| Mutation (+ve) | t_15-55_ | 0 (0.0) | 1 (3.3) | 0.27 |
| Wild-type (-ve) |  | 2 (2.2) | 1 (3.3) |  |
| ^ba^CBFB, n (%) |  |  |  |  |
| Mutation (+ve) | t_0_ | 9 (9.9) | 0 (0.0) | 4.01 |
| Wild-type (-ve) |  | 72 (79.1) | 0 (0.0) |  |
| Mutation (+ve) | t_15-55_ | 2 (2.2) | 0 (0.0) | 0.22 |
| Wild-type (-ve) |  | 4 (4.4) | 1 (3.3) |  |
| ^ba^NPM1, n (%) |  |  |  |  |
| Mutation (+ve) | t_0_ | 25 (27.5) | 2 (6.7) | 2.57 |
| Wild-type (-ve) |  | 53 (58.2) | 23 (76.7) |  |
| Mutation (+ve) | t_15-55_ | 2 (2.2) | 0 (0.0) | 0.26 |
| Wild-type (-ve) |  | 1 (1.1) | 1 (3.3) |  |
| ^ba^IDH2, n (%) |  |  |  |  |
| Mutation (+ve) | t_0_ | 14 (15.4) | 3 (10.0) | 0.17 |
| Wild-type (-ve) |  | 64 (70.3) | 22 (73.3) |  |
| Mutation (+ve) | t_15-55_ | 3 (3.3) | 0 (0.0) | 0.15 |
| Wild-type (-ve) |  | 1 (1.1) | 1 (3.3) |  |
| Fluorescence in situ hybridization (FISH), n (%) |  |  |  |  |
| ^ba^EGR1 |  |  |  |  |
| Mutation (+ve) | t_0_ | 13 (16.5) | 0 (0.0) | 3.81 |
| Wild-type (-ve) |  | 67 (73.6) | 0 (0.0) |  |
| Mutation (+ve) | t_15-55_ | 6 (6.6) | 0 (0.0) | 0.60 |
| Wild-type (-ve) |  | 8 (8.8) | 0 (0.0) |  |
| ^ba^MLL |  |  |  |  |
| Mutation (+ve) | t_0_ | 15 (16.5) | 0 (0.0) | 3.18 |
| Wild-type (-ve) |  | 61 (67.0) | 0 (0.0) |  |
| Mutation (+ve) | t_15-55_ | 8 (8.8) | 0 (0.0) | 0.55 |
| Wild-type (-ve) |  | 4 (4.4) | 0 (0.0) |  |
| ^ba^8Centromere |  |  |  |  |
| Mutation (+ve) | t_0_ | 20 (22.0) | 0 (0.0) | 3.01 |
| Wild-type (-ve) |  | 55 (60.4) | 0 (0.0) |  |
| Mutation (+ve) | t_15-55_ | 12 (13.2) | 0 (0.0) | 0.80 |
| Wild-type (-ve) |  | 10 (11.0) | 0 (0.0) |  |

**^a^**Summary statistics (i.e., median, IQR, range, *P* value, standardized mean difference (SMD)) were calculated.

**^b^**Features used in ML models.

**Supplementary Table 9. Summary of post treatment events among AML patients in ven/aza training and external validation group.**

|  | Time | Training | Validation | SMD |
| --- | --- | --- | --- | --- |
| N |  | 91 | 30 |  |
| Transfusions, Median (IQR) [Range] |  |  |  |  |
| ^b^Platelet bags, Count | t_ind_ | 4.1 (8.4) | 4.3 (4.7) | 0.03 |
| ^b^Red blood cell bags, Count | t_ind_ | 4.1 (3.4) | 3.8 (3.1) | 0.09 |
| ^b^Length of stay, Median (IQR) [Range], Days | t_ind_ | 9.8 (4.9) | 7.5 (5.1) | 0.45 |
| ^b^ICU transfer during induction, n (%) | t_ind_ | 5 (5.5) | 1 (3.3) | 0.11 |
| Toxicity, n (%) |  |  |  |  |
| ^b^ALT | t_<30_ |  |  |  |
| ≥Grade 1 |  | 16 (17.6) | 9 (30.0) | 0.33 |
| Non-toxic |  | 74 (81.3) | 21 (70.0) |  |
| ^b^AST ≥Grade 2 | t_<30_ | 4 (4.4) | 3 (10.0) | 0.22 |
| ^b^Bilirubin ≥Grade 2 | t_<30_ | 15 (16.5) | 4 (13.3) | 0.09 |
| ^b^Anemia ≥Grade 3 | t_<30_ | 82 (90.1) | 26 (86.7) | 0.11 |
| ^b^Chronic kidney disease | t_<30_ |  |  |  |
| ≥Grade 3 |  | 6 (6.6) | 2 (6.7) | 1.32 |
| ≤Grade 2 |  | 25 (27.5) | 24 (80.0) |  |
| ^b^Creatinine ≥Grade 2 | t_<30_ | 10 (11.1) | 3 (10.0) | 0.03 |
| ^b^Ejection fraction | t_<30_ |  |  |  |
| ≥Grade 1 |  | 3 (3.3) | 1 (3.3) | 0.25 |
| Non-toxic |  | 15 (16.5) | 8 (26.7) |  |
| ^b^Neutrophils ≥Grade 4 | t_<30_ | 88 (96.7) | 27 (90.0) | 0.27 |
| ^b^Proteinuria | t_<30_ |  |  |  |
| ≥Grade 2 |  | 13 (14.3) | 5 (16.7) | 0.26 |
| ≤Grade 1 |  | 48 (52.7) | 12 (40.0) |  |
| ^b^Thrombocytopenia |  |  |  |  |
| ≥Grade 4 |  | 61 (67.0) | 21 (70.0) | 0.06 |
| Readmission or occurrences after discharge, n (%) |  |  |  |  |
| ^b^Hospital readmission by 30d FUP Bx date | t_<30_ | 26(28.6) | 10 (33.3) | 0.10 |
| ^b^ICU readmission by 30d FUP Bx date | t_<30_ | 2 (2.2) | 0 (0.0) | 0.21 |
| ^b^At least one bag of RBC transfusion by 30d FUP Bx date | t_<30_ | 20 (22.0) | 10 (33.3) | 0.26 |
| ^b^At least one bag of platelet transfusion by 30d FUP Bx date | t_<30_ | 16 (17.6) | 5 (16.7) | 0.02 |
| ^b^Follow-up biopsy 30d responses, n (%) |  |  |  | 0.14 |
| Complete remission | t_15-55_ | 9 (9.9) | 2 (6.7) |  |
| Complete remission with incomplete hematologic recovery | t_15-55_ | 39 (42.9) | 13 (43.3) |  |
| Morphological leukemia-free state | t_15-55_ | 22 (24.2) | 7 (23.3) |  |
| Stable disease | t_15-55_ | 21 (23.1) | 8 (26.7) |  |
| ^b^Date differences between TX start date and 30d FUP Bx date, n (%) | t_15-55_ |  |  | 0.33 |
| [15d,25d] | t_15-55_ | 14 (15.4) | 8 (26.7) |  |
| (25d,30d] | t_15-55_ | 71 (78.0) | 19 (63.3) |  |
| (30d,55d] | t_15-55_ | 6 (6.6) | 3 (10.0) |  |

**^b^**Features used in ML models.

**Supplemental Table 10. Definition of ejection fraction (EF) toxicity grades.**

| Grade | Definition |
| --- | --- |
| 4 | Follow Up EF Value < 20.00% |
| 3 | Follow Up EF Value lies between [20,40)% or  The difference between (Treatment Start EF Value - Follow Up EF value)  ≥ 20% |
| 2 | Follow Up EF Value between [40,50)% or  The difference between (Treatment Start EF Value - Follow Up EF value) between [10,20)% |

**Supplemental Table 11. Definition of features signaling genetic abnormality.**

| Genetic abnormality | FISH | PCR | NGS |
| --- | --- | --- | --- |
| TP53 | Y |  | Y |
| NPM1 |  | Y | Y |
| CBFB | Y | Y |  |
| PML | Y | Y | Y |
| RUNX | Y | Y | Y |
| KMT2A(MLL)/EFR1 | Y | Y |  |
| Flt3 |  | Y | Y |
| IDH1 and IDH2 |  | Y | Y |
| EGR1 | Y | Y |  |

**Supplemental Table 12. List of features used in developing predictive models.**

| **Patient-specific features** | |
| --- | --- |
| Age | **Numeric** |
| Ethnicity | **Categorical** |
| Gender | **Categorical** |
| Obesity | **Categorical** |
| Albumin (g/dL) | **Numeric (day0 and day30)** |
| ANC (10^9^/L) | **Numeric (day0 and day30)** |
| AST (U/L) | **Numeric (day0 and day30)** |
| Bilirubin (mg/dL) | **Numeric (day0 and day30)** |
| Calcium (mg/dL) | **Numeric (day0 and day30)** |
| Creatinine (mg/dL) | **Numeric (day0 and day30)** |
| Fibrinogen (mg/dL) | **Numeric (day0 and day30)** |
| Hemoglobin (g/dL) | **Numeric (day0 and day30)** |
| LDH (U/L) | **Numeric (day0 and day30)** |
| Lymphocytes (10^9^/L) | **Numeric (day0 and day30)** |
| Phosphorus (mg/dL) | **Numeric (day0 and day30)** |
| Platelets (10^9^/L) | **Numeric (day0 and day30)** |
| Potassium (mmol/L) | **Numeric (day0 and day30)** |
| Uric-acid (mg/dL) | **Numeric (day0 and day30)** |
| WBC (10^9^/L) | **Numeric (day0 and day30)** |
| Blasts (%) | **Numeric (day0 and day30)** |
| CD7 | **Categorical (day0 and day30)** |
| CD117 | **Categorical (day0 and day30)** |
| CD11B | **Categorical (day0 and day30)** |
| CD123 | **Categorical (day0 and day30)** |
| CD13 | **Categorical (day0 and day30)** |
| CD14 | **Categorical (day0 and day30)** |
| CD15 | **Categorical (day0 and day30)** |
| CD33 | **Categorical (day0 and day30)** |
| CD34 | **Categorical (day0 and day30)** |
| CD38 | **Categorical (day0 and day30)** |
| CD45 | **Categorical (day0 and day30)** |
| CD56 | **Categorical (day0 and day30)** |
| CD64 | **Categorical (day0 and day30)** |
| HLADR | **Categorical (day0 and day30)** |
| MPO | **Categorical (day0 and day30)** |
| EGR1 | **Categorical (day0 and day30)** |
| MLL | **Categorical (day0 and day30)** |
| 7Centromere | **Categorical (day0 and day30)** |
| 8Centromere | **Categorical (day0 and day30)** |
| Cytogenetics | **Poor, Indeterminant, Intermediate, Good (day0 and day30)** |
| CBFB | **Categorical (day0 and day30)** |
| FLT3 | **Categorical (day0 and day30)** |
| IDH2 | **Categorical (day0 and day30)** |
| NPM1 | **Categorical (day0 and day30)** |
| RUNX | **Categorical (day0 and day30)** |

| **Clinical events occurred during initial hospitalization, after discharge, and within first 30d** | |
| --- | --- |
| ICU transfer | **Categorical** |
| Hospitalization during initial induction period (days) | **Numeric** |
| RBC transfusions during initial admission (bags) | **Numeric** |
| Platelet transfusions during initial admission (bags) | **Numeric** |
| FUP Response at 30d | **Categorical** |
| Difference between TX start date & 30d FUP Bx date | **Numeric** |
| Hospital re-admission | **Categorical** |
| Hospital ICU re-transfer after discharge & before 30d FUP response | **Categorical** |
| RBC transfusions after discharge & before 30d FUP response (bags) | **Numeric** |
| Platelet transfusions after discharge & before 30d FUP response (bags) | **Numeric** |
| Creatinine toxicity within first 30d of treatment start (CTCAE) | **Ordinal** |
| CKD within first 30d of treatment start (CTCAE) | **Ordinal** |
| Bilirubin toxicity within first 30d of treatment start (CTCAE) | **Ordinal** |
| AST toxicity within first 30d of treatment start (CTCAE) | **Ordinal** |
| ALT toxicity within first 30d of treatment start (CTCAE) | **Ordinal** |
| Proteinuria within first 30d of treatment start (CTCAE) | **Ordinal** |
| Ejection fraction within first 30d of treatment start (CTCAE) | **Ordinal** |
| Anemia toxicity within first 30d of treatment start (CTCAE) | **Ordinal** |
| Thrombocytopenia within first 30d of treatment start (CTCAE) | **Ordinal** |
| Neutrophils within first 30d of treatment start (CTCAE) | **Ordinal** |
| Febrile neutropenia toxicity within first 30d of treatment start (CTCAE) | **Ordinal** |

**Supplemental Table 13. Features associated with overall survival based on multivariate models.** Orange and Green color shades correspond to the negative (worse/”W”) and positive (better/”B”) association, respectively. “NA” corresponds to not available as the corresponding variable was not added in the multivariate model due to zero-variation or high missingness. “N” corresponds to neutrality.

|  | 7+3 | Ven/aza |
| --- | --- | --- |
| Age > 75 | W | W |
| Obesity | W | N |
| Prior MDS | N | W |
| Prior coagulopathy | W | W |
| Prior hypertension | W | N |
| Abnormal WBC | W | W |
|  | W | N |
| Blast > 20% | N | W |
|  | W | W |
| Abnormal HGB | N | W |
|  | W | N |
| Abnormal PLT | W | W |
|  | W | W |
| Abnormal CRT | W | W |
|  | N | W |
| Abnormal Uric-acid | W | W |
|  | W | NA |
| Abnormal AST | W | W |
|  | W | W |
| Abnormal LDH | W | W |
|  | N | W |
| Abnormal albumin | W | W |
|  | W | W |
| ELN Adverse | W | N |
| Cytogenetics: good | B | N |
|  | NA | NA |
| Cytogenetics: Poor | W | W |
|  | W | W |
| CD117 | B | N |
|  | N | W |
| CD11B | N | W |
|  | N | W |
| CD13 | B | N |
|  | W | N |
| CD123 | B | N |
|  | N | N |
| CD33 | B | N |
|  | N | N |
| CD34 | N | W |
|  | N | N |
| CD38 | B | W |
|  | N | N |
| MPO | B | B |
|  | N | N |
| CD7 | N | W |
|  | N | W |
| CD64 | N | N |
|  | N | W |
| 7centromere | N | N |
|  | W | W |
| 8centromere | N | N |
|  | W | N |
| EGR1 | W | N |
|  | W | W |
| CBFB | B | W |
|  | N | NA |
| FLT3 | N | W |
|  | W | NA |
| IDH2 | B | B |
| MLL | W | N |
| NPM1 | W | B |
| RUNX | W | N |
| ICU transfer | W | N |
| High LOS during induction | W | W |
| PLT transfusion > 5 | W | N |
| Thrombocytopenia > Grade 3 | NA | W |
| Creatinine > Grade 1 | W | N |
| Anemia > Grade 2 | NA | W |
| AST > Grade 1 | N | W |
| CKD > Grade 2 | W | W |
| Ejection fraction | W | W |
| Follow-up response: CR/CRi | B | B |
| Follow-up response: SD | N | W |

**SUPPLEMENTAL FIGURE LEGENDS**

**Supplemental Figure 1. Summary of patient numbers (left) and the corresponding analyses performed (right).**

**Supplemental Figure 2. Adjusted hazard ratios (aHRs) for predictors of overall survival for the ven/aza cohort corresponding to diagnostic features.** Reported are the aHRs (vertical tick) and bootstrap based 95% confidence intervals (horizontal line). “Reference features” correlating with a better outcome are to the right and “Label features” with a better outcome are to the left. The number of patients who died relative to the subset of patients with each feature is summarized at the far left. The table includes baseline demographics, disease characteristics, and comorbidities.

**Supplemental Figure 3. Univariate analysis of Day_15-55_ response assessment and long-term outcomes (7+3 left, ven/aza right).** CR/CRi vs other responses for all patients (top), CR/CRi vs other responses excluding patients who received an allogeneic SCT (middle), and CR/CRi vs MLFS for all patients (bottom). As described in the methods section, Day_15-55_ is defined as a bone marrow biopsy and other clinical evaluation done within 15 days to 55 days from initiation of treatment and closest to Day 30.

**Supplemental Figure 4. Adjusted hazard ratio for 7+3 treated patients.** Forest plot showing the aHRs (vertical tick) and bootstrap based 95% confidence intervals (horizontal line). Reference features” correlating with a better outcome is to the right and “Label features” with a better outcome are to the left. The number of patients who died relative to the subset of patients with each feature (out of the total cohort of 111 patients) is at the far left. Baseline demographic and disease characteristics and comorbidities are at the top of the table, events occurring during the first 30 days are in the mid-section and outcomes at the day_15-55_ bone marrow biopsy and blood testing are in the bottom section.

**Supplemental Figure 5. Univariate analysis of the impact of clinical events occurring during first 30 days of therapy and long-term outcomes.** (7+3 left, ven/aza right). A) AST during first 30 days of therapy; B) Creatinine during first 30 days of therapy; C) Onset of ≥Grade 2 CKD; D) Onset of poor (≥Grade 1) ejection fraction; E) Febrile neutropenia during first 30 days of therapy; F) Onset of ≥Grade 4 neutrophils and ≥Grade 3 febrile neutropenia; G) Platelet transfusions during induction period; H) Red blood cell transfusions during induction period; I) ICU transfer during induction period.

**Supplemental Figure 6. Machine learning based prospective modeling for** a ven/aza treated patient.

**Supplemental Figure 7. Machine learning based prospective modeling for** a 7+3 treated patient.

**Supplemental Figure 8. Disease state transitions within a year,**

**Supplemental Figure 9. Disease state transition probabilities between 90-365 days**, Heat map of the probabilities of transitioning from a disease state (Y-axis legends) at day 90 (secondary Y-axis legends) to other disease states at day 365 (X-axis legends). Probabilities are in the left heat map and standard errors in the right heat map.

**Supplemental Figure 10. Disease state transition probabilities between 180-365 days**. Heat map of the probabilities of transitioning from a disease state (Y-axis legends) at day 90 (secondary Y-axis legends) to other disease states at day 365 (X-axis legends). Probabilities are in the left heat map and standard errors in the right heat map.

**Supplemental Figure 1. Counts of patients in the analytical datasets and associated analyses.**

Number of newly diagnosed AML patients in 7+3 and Ven/Aza cohort

N_7+3_ = 120 and N_v/a_ = 101

Number of AML patients with valid follow-up (post-TX) bone marrow biopsy

N_7+3_ = 115 and N_v/a_ = 98

Number of AML patients with 30d (responses were recorded within 15d-55d post-TX) follow-up bone marrow biopsy

N_7+3_ = 111 and N_v/a_ = 91

-Kaplan-Meier (Figure 1, Supplemental Figure 1A-C) and cumulative hazard (time-to-onset) analyses related to toxicity variables (Supplemental Figure 4)

-Kaplan-Meier analyses related to 30d responses (e.g., follow-up responses, 30d blasts) (Figure 1)

-Adjusted hazard ratios estimation based on penalized regression (Table 1, Supplemental Table 2)

-Machine-learning based prospective modeling (Table 2, Figure 2, Supplemental Figure 5, Supplemental Table 3)

-Summary measures (Supplemental Table 1, 4)

-Estimation of transition probabilities and occupation probabilities based on multistate survival analyses (Figure 4, Supplemental Figure 4)

Number of AML patients with “best” response associated with each treatment (before the administration of any subsequent therapy (e.g., HiDAC, transplant)) in 7+3 and ven/aza cohort

N_7+3_ = 118 and N_v/a_ = 101

-KM for “Best” response to each treatment (Figure 3)

**Supplemental Figure 2. Adjusted hazard ratios (aHRs) for predictors of overall survival for the ven/aza cohort corresponding to diagnostic features.**


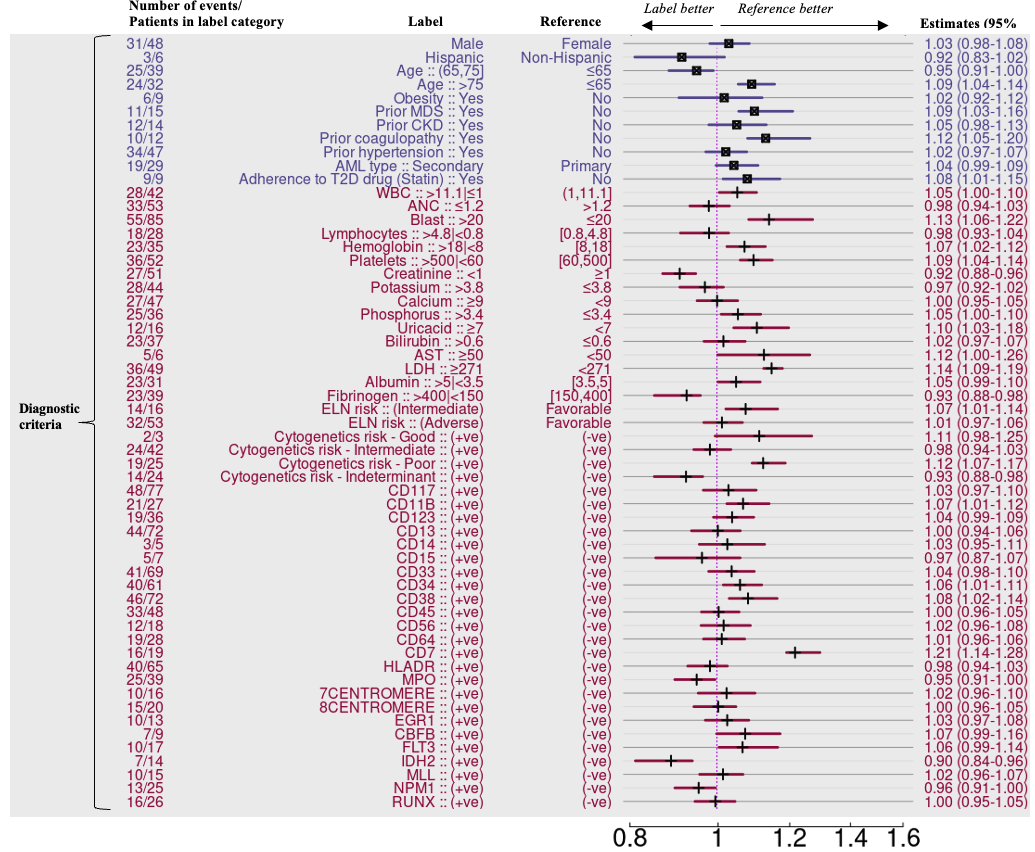


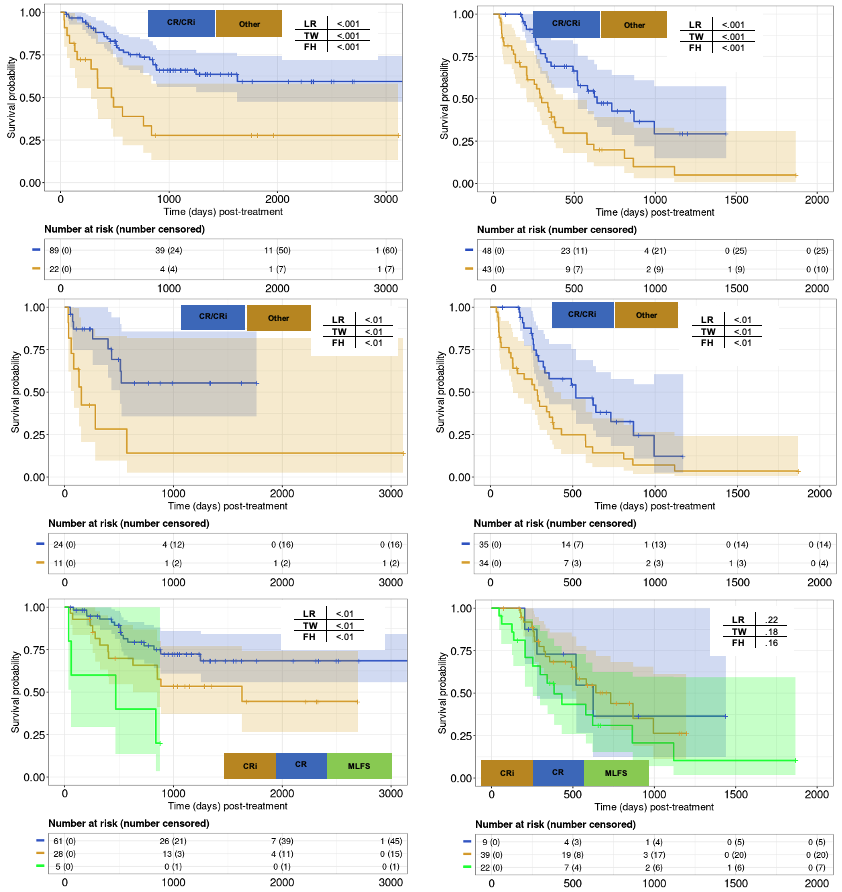
**Supplemental Figure 3. Univariate analysis of Day_15-55_ response assessment and long-term outcomes (7+3 left, ven/aza right).** CR/CRi vs other responses for all patients (top), CR/CRi vs other responses excluding patients who received an allogeneic SCT (middle), and CR/CRi vs MLFS for all patients (bottom). As described in the methods section, Day_15-55_ is defined as a bone marrow biopsy and other clinical evaluation done within 15 days to 55 days from initiation of treatment and closest to Day 30.

**Supplemental Figure 4. Adjusted hazard ratios for confounders of overall survival for 7+3 cohort.**


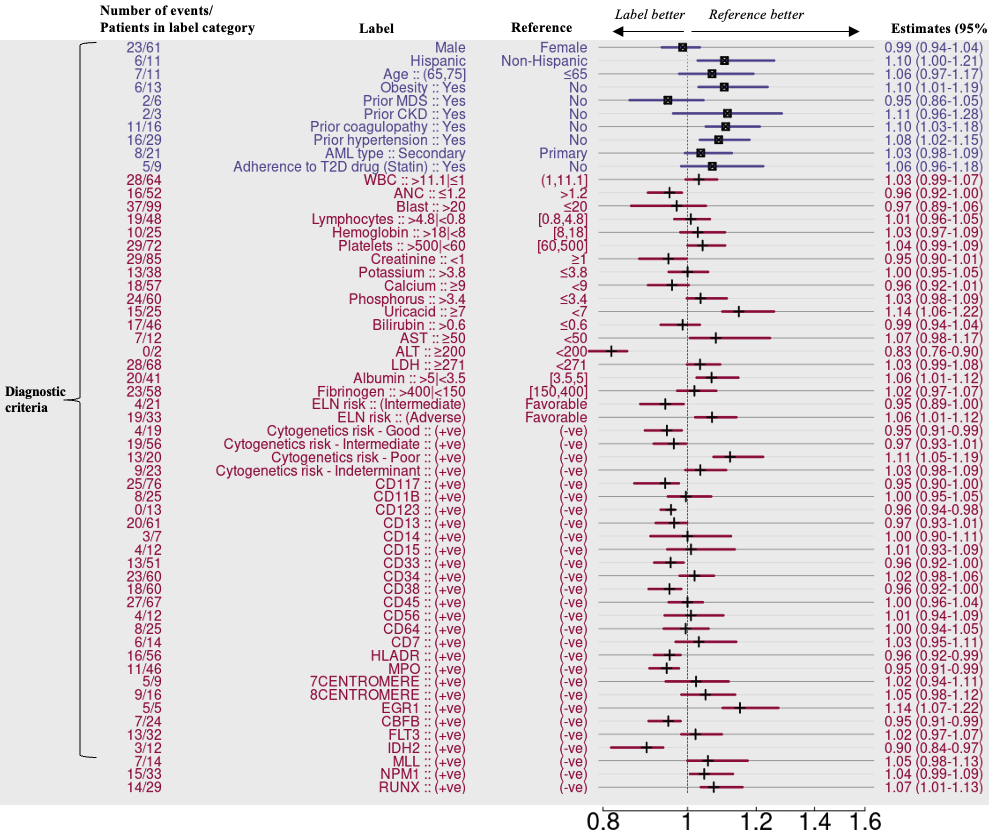


**
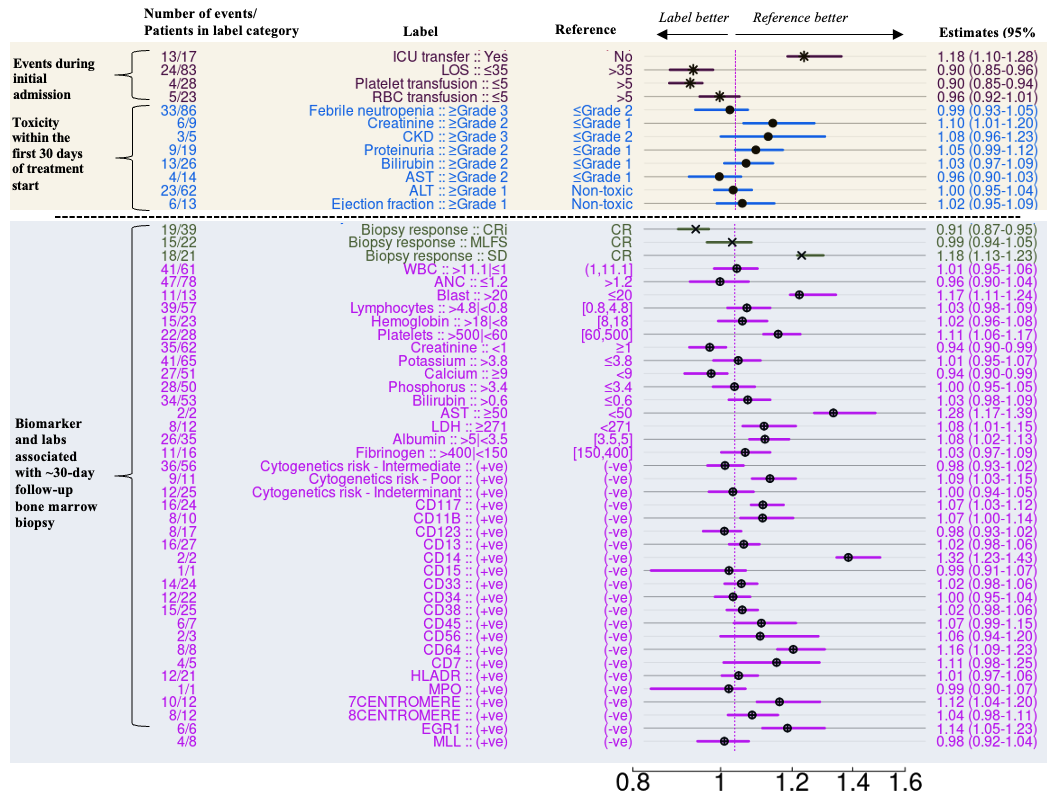
**

**Supplemental Figure 5. Univariate analysis of the impact of toxicities and clinical events occurring during first 30 days of therapy and long-term outcomes (7+3 left, ven/aza right).**

1. **AST during first 30 days of therapy**


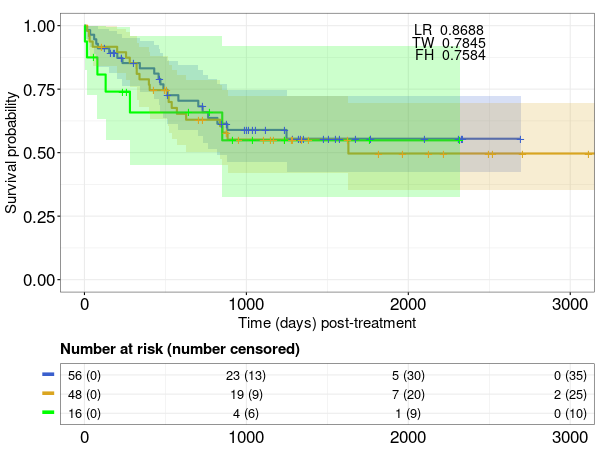


| **LR** | .87 |
| --- | --- |
| **TW** | .79 |
| **FH** | .76 |


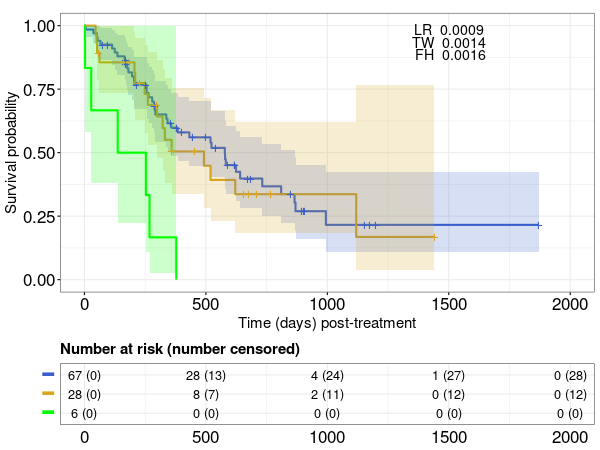


| **LR** | <.001 |
| --- | --- |
| **TW** | <.01 |
| **FH** | <.01 |


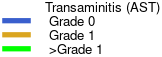


1. **Creatinine during first 30 days of therapy**


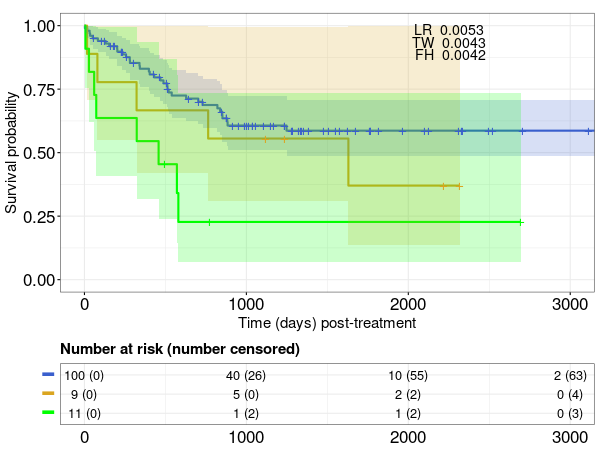


| **LR** | <.01 |
| --- | --- |
| **TW** | <.01 |
| **FH** | <.01 |


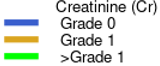

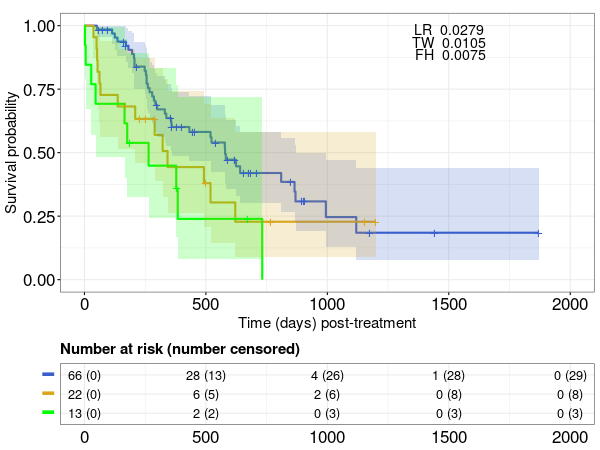


| **LR** | .03 |
| --- | --- |
| **TW** | .01 |
| **FH** | <.01 |

1. **Onset of ≥Grade 2 CKD**


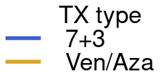

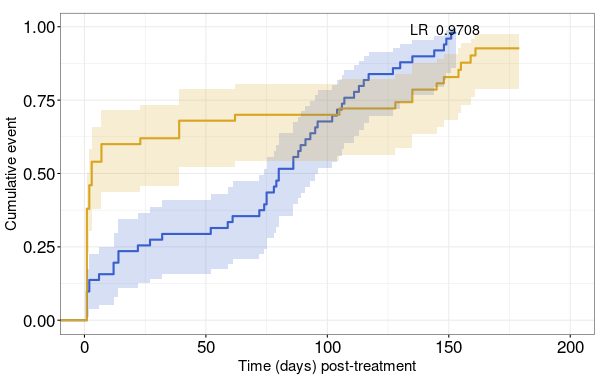


| **LR** | .97 |
| --- | --- |

1. **Onset of poor (≥Grade 1) ejection fraction**


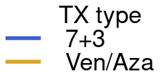

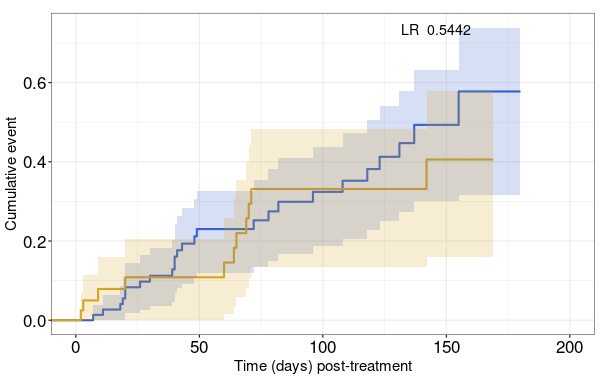


| **LR** | .54 |
| --- | --- |

1. **Febrile neutropenia during first 30 days of therapy**


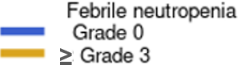

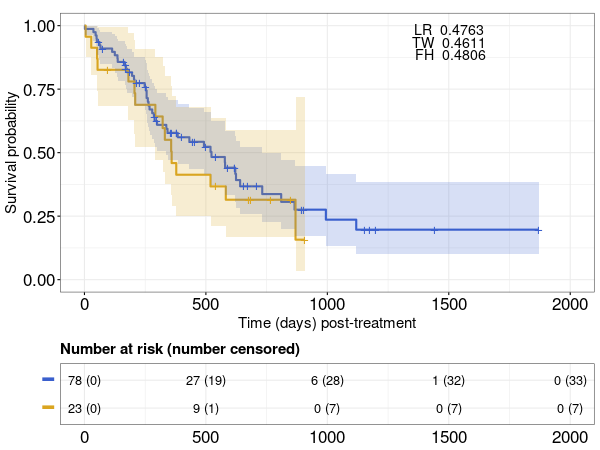

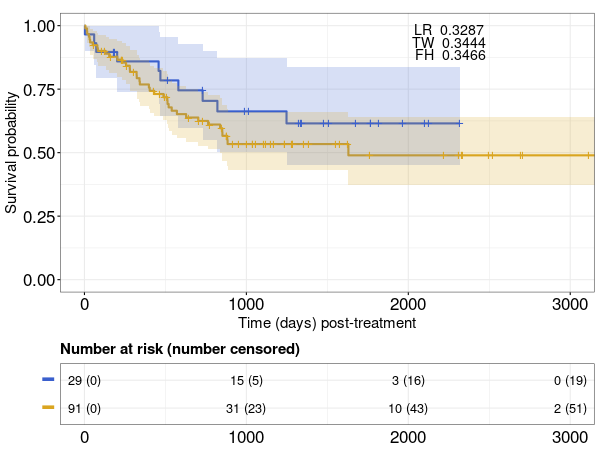


| **LR** | .33 |
| --- | --- |
| **TW** | .34 |
| **FH** | .35 |

| **LR** | .48 |
| --- | --- |
| **TW** | .46 |
| **FH** | .48 |

1. **Onset of ≥Grade 4 neutrophils and ≥Grade 3 febrile neutropenia**


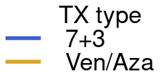

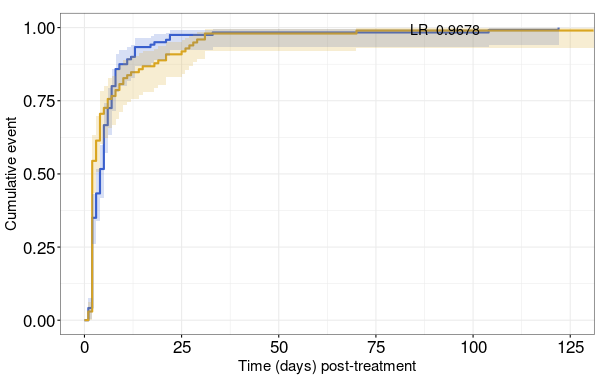

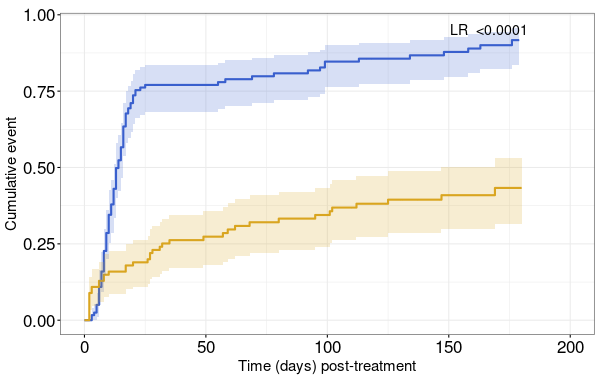


| **LR** | <.001 |
| --- | --- |

| **LR** | .97 |
| --- | --- |

1. **Platelet transfusions during induction period**


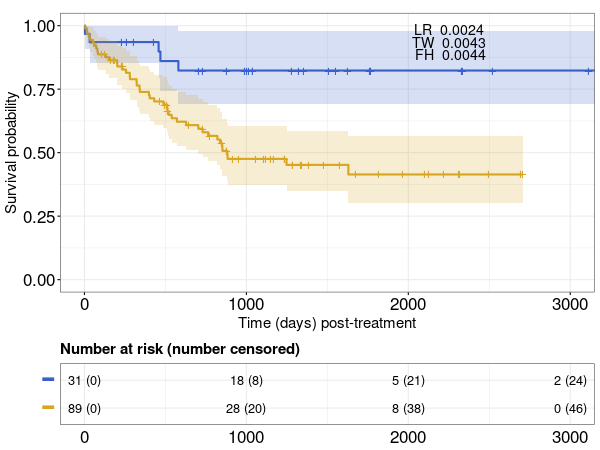

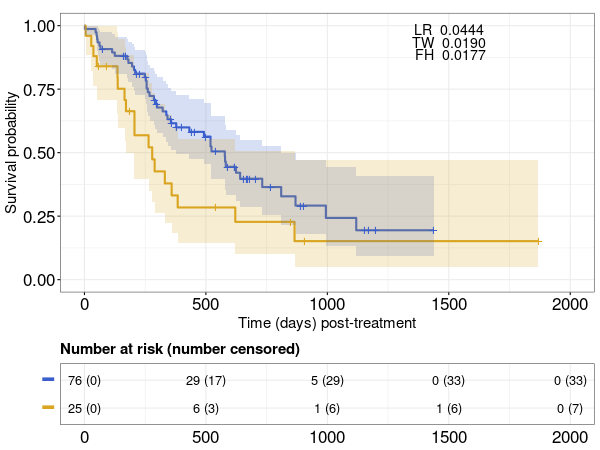


| **LR** | <.01 |
| --- | --- |
| **TW** | <.01 |
| **FH** | <.01 |

| **LR** | <.01 |
| --- | --- |
| **TW** | <.01 |
| **FH** | <.01 |


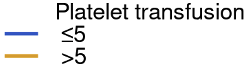


**H. Red blood cell transfusions during induction period**


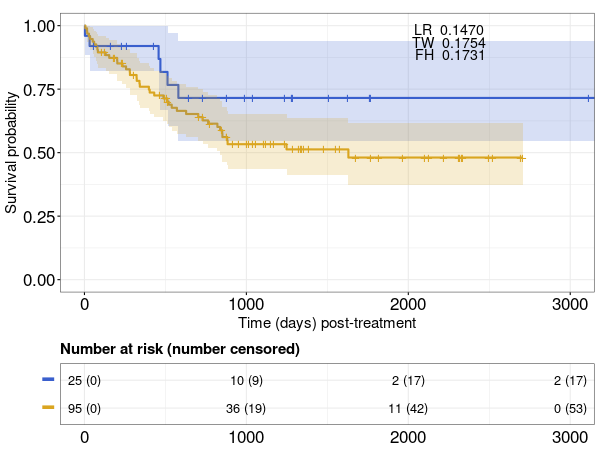

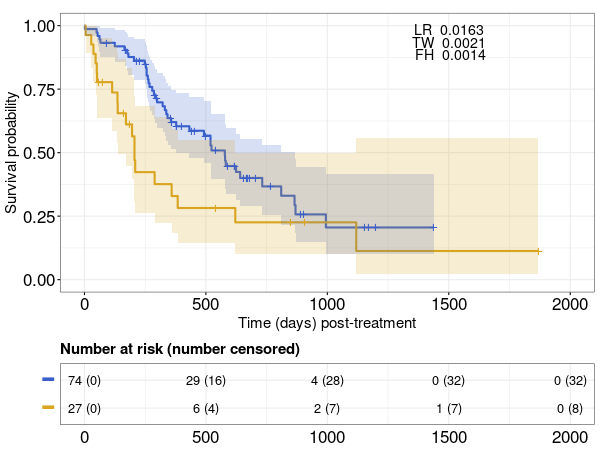


| **LR** | .02 |
| --- | --- |
| **TW** | <.01 |
| **FH** | <.01 |

| **LR** | .15 |
| --- | --- |
| **TW** | .18 |
| **FH** | .17 |


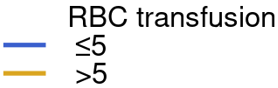


1. **ICU transfer during induction period**


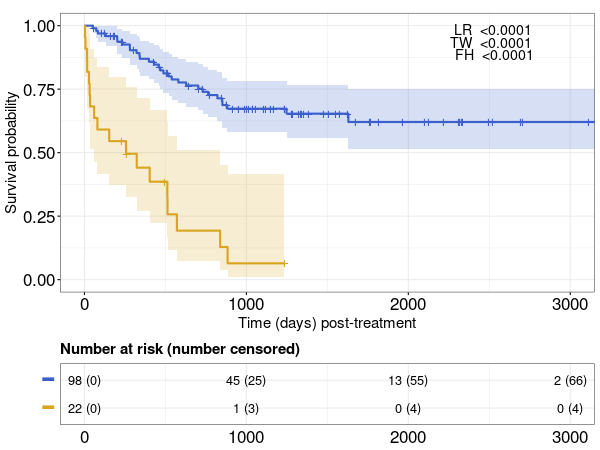


| **LR** | <.001 |
| --- | --- |
| **TW** | <.001 |
| **FH** | <.001 |


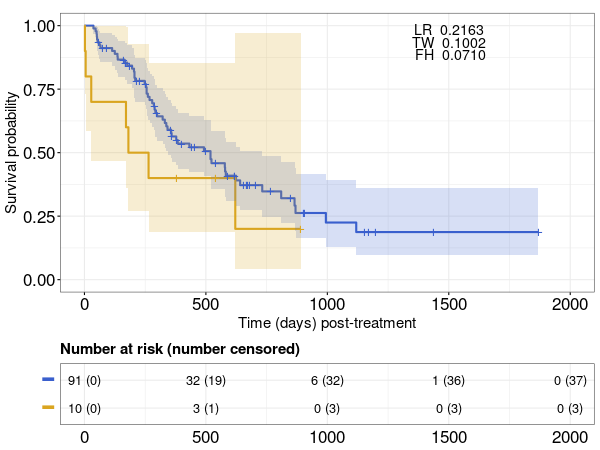

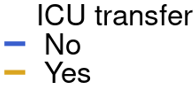


| **LR** | .21 |
| --- | --- |
| **TW** | .10 |
| **FH** | .07 |

**Supplemental Figure 6. Representative ML predictions for a patient treated with ven/aza occurring during the first 30 days of treatment and the and Day_15-55_ assessment.**

| **Demographic** | |
| --- | --- |
| ^b^Age | **74** |
| ^b^Ethnicity | **Hispanic** |
| ^b^Gender | **Male** |

| **Labs** | **t_0_** | **t_15-55_** | **Pathology** | **t_0_** | **t_15-55_** | **Comorbidities** | |
| --- | --- | --- | --- | --- | --- | --- | --- |
| ^b^Albumin (g/dL) | **3.4** | **3.2** | ^b^*Blasts (%)* | **94.0** | **7.0** | *Coagulopathy* | **Yes** |
| ^b^ANC (10^9^/L) | **3.3** | **0.1** | ^b^*CD7* | **(-ve)** | **(-ve)** | *MDS* | **No** |
| ^b^AST (U/L) | **37.0** | **17.0** | *^b^CD117* | **(+ve)** | **(+ve)** | ^b^*Obesity* | **No** |
| ^b^Bilirubin (mg/dL) | **0.4** | **0.3** | ^b^*CD11B* | **(+ve)** | **(-ve)** | *AML type* | **Primary** |
| ^b^Calcium (mg/dL) | **8.2** | **8.8** | ^b^*CD123* | **(-ve)** | **(-ve)** | *Non-AML cancer* | **No** |
| ^b^Creatinine (mg/dL) | **0.9** | **1.0** | ^b^*CD13* | **(+ve)** | **(-ve)** | *Treatment related AML* | **Yes** |
| ^b^Fibrinogen (mg/dL) | **216.0** | **296.0** | ^b^*CD14* | **(-ve)** | **(-ve)** |  |  |
| ^b^Hemoglobin (g/dL) | **9.9** | **9.2** | ^b^*CD15* | **(-ve)** | **(-ve)** |  |  |
| ^b^LDH (U/L) | **1116.0** | **145.0** | ^b^*CD33* | **(+ve)** | **(-ve)** |  |  |
| ^b^Lymphocytes (10^9^/L) | **10.0** | **0.5** | ^b^*CD34* | **(-ve)** | **(-ve)** |  |  |
| ^b^Phosphorus (mg/dL) | **1.4** | **4.0** | ^b^*CD38* | **(+ve)** | **(+ve)** |  |  |
| ^b^Platelets (10^9^/L) | **18.0** | **165.0** | ^b^*CD45* | **(+ve)** | **(+ve)** |  |  |
| ^b^Potassium (mmol/L) | **4.5** | **4.3** | ^b^*CD56* | **(-ve)** | **(-ve)** |  |  |
| ^b^Uric-acid (mg/dL) | **4.8** | **4.8** | ^b^*CD64* | **(+ve)** | **(-ve)** |  |  |
| ^b^WBC (10^9^/L) | **166.9** | **0.6** | ^b^*HLADR* | **(-ve)** | **(-ve)** |  |  |
|  |  |  | ^b^*MPO* | **(+ve)** | **(-ve)** |  |  |
|  |  |  | ^b^*EGR1* | **(-ve)** | **(NP)** |  |  |
|  |  |  | ^b^*MLL* | **(-ve)** | **(NP)** |  |  |
|  |  |  | ^b^*7Centromere* | **(-ve)** | **(NP)** |  |  |
|  |  |  | ^b^*8Centromere* | **(-ve)** | **(NP)** |  |  |
|  |  |  | ^b^*Cytogenetics* | **Intermediate** | **Intermediate** |  |  |
|  |  |  | *ELN risk* | **Intermediate** | **Intermediate** |  |  |
|  |  |  | ^b^*CBFB* | **(-ve)** | **(NP)** |  |  |
|  |  |  | ^b^*FLT3* | **(+ve)** | **+ve)** |  |  |
|  |  |  | ^b^*IDH2* | **(-ve)** | **(-ve)** |  |  |
|  |  |  | ^b^*NPM1* | **(+ve)** | **(+ve)** |  |  |
|  |  |  | ^b^*RUNX* | **(-ve)** | **(-ve)** |  |  |

**Venetoclax + Azacitidine**

| **TX related side effects, t_<30_** | |
| --- | --- |
| ^b^Creatinine toxicity (CTCAE) | **G1** |
| ^b^CKD (CTCAE) | **G1** |
| ^b^Bilirubin toxicity (CTCAE) | **Non-toxic** |
| ^b^AST toxicity (CTCAE) | **G3** |
| ^b^ALT toxicity (CTCAE) | **G1** |
| ^b^Proteinuria (CTCAE) | **Unknown** |
| ^b^Ejection fraction (CTCAE) | **G3** |
| ^b^Anemia toxicity (CTCAE) | **G3** |
| ^b^Thrombocytopenia (CTCAE) | **G4** |
| ^b^Neutrophils (CTCAE) | **G4** |
| ^b^Febrile neutropenia toxicity (CTCAE) | **Non-toxic** |

| **Responses by 30d FUP Bx date, t_<30_** | |
| --- | --- |
| ^b^FUP Response at 30d | **SD** |
| ^b^Difference between TX start date & 30d FUP Bx date | **(25,30]** |
| ^b^Hospital re-admission | **No** |
| ^b^Hospital ICU re-transfer | **No** |
| ^b^RBC transfusions (bags) | **0** |
| ^b^Platelet transfusions (bags) | **0** |
| Stem-cell transplant | **No** |

| **Long term responses** | |
| --- | --- |
| Survival status | **Deceased** |
| Time-to-censor (days) | **137** |

| **Induction related responses, t_ind_** | |
| --- | --- |
| ^b^ICU transfer | **No** |
| ^b^Hospitalization (days) | **29** |
| ^b^RBC transfusions (bags) | **6** |
| ^b^Platelet transfusions (bags) | **10** |


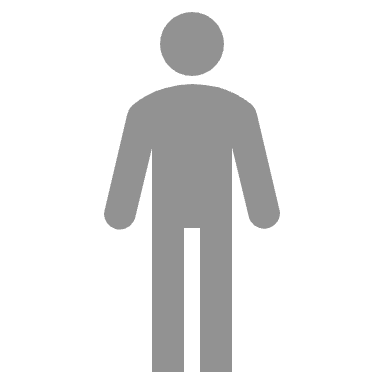

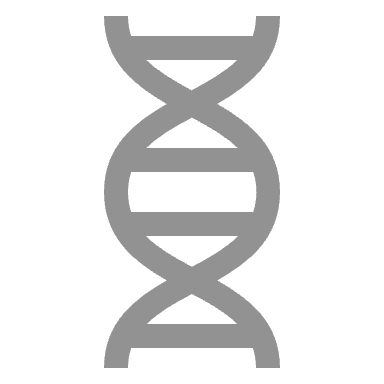

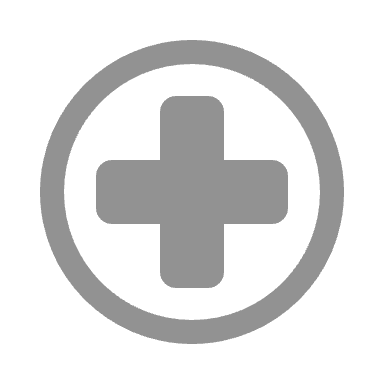

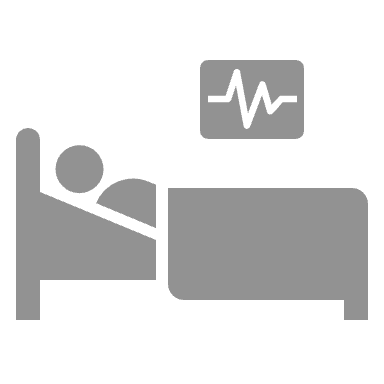

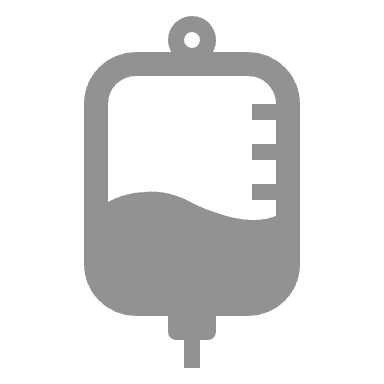

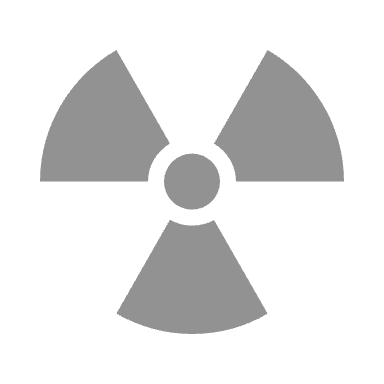

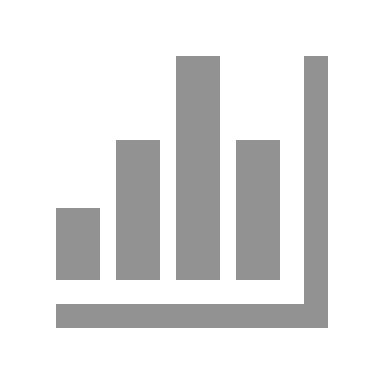


Remark: ^b^Features used in ML model

**Overall survival up to 2 years**

| **7+3** | **Venetoclax + Azacitidine** |
| --- | --- |

**KM probability at (365d,730d) = (0.82,0.67)**

**Training model cAUC: 1.00 || C_365_: 0.88 || Brier_365_: 0.03**

**KM probability at (365d,730d) = (0.56,0.35)**

**Training model cAUC: 0.96 || C_365_: 0.87 || Brier_365_: 0.13**


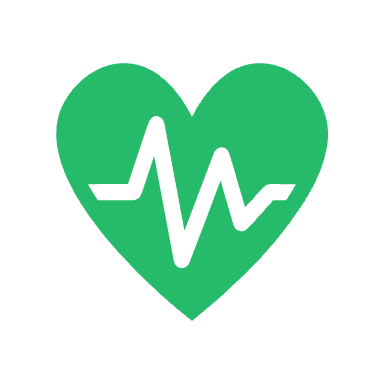

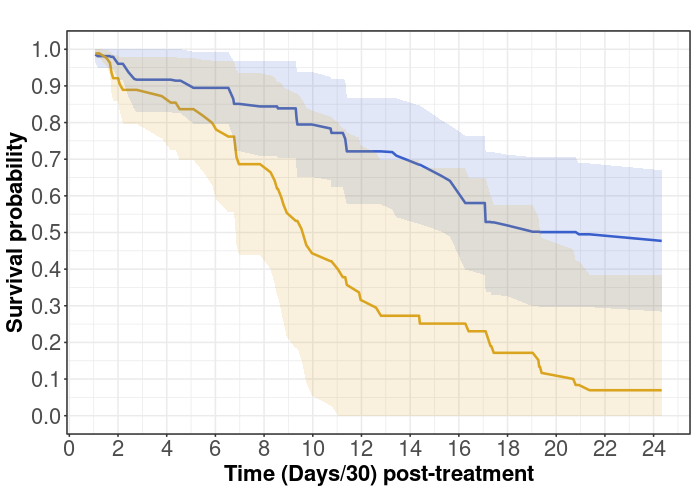


**Supplemental Figure 7. Representative ML predictions for a patient treated with 7+3 occurring during the first 30 days of treatment and the and Day_15-55_ assessment**

| **Demographic** | |
| --- | --- |
| ^b^Age | **70** |
| ^b^Ethnicity | **Non-Hispanic** |
| ^b^Gender | **Male** |

| **Labs** | **t_0_** | **t_15-55_** | **Pathology** | **t_0_** | **t_15-55_** | **Comorbidities** | |
| --- | --- | --- | --- | --- | --- | --- | --- |
| ^b^Albumin (g/dL) | **3.0** | **3.7** | ^b^*Blasts (%)* | **49.5** | **2.5** | *Coagulopathy* | **No** |
| ^b^ANC (10^9^/L) | **0.0** | **1.3** | ^b^*CD7* | **(-ve)** | **(-ve)** | *MDS* | **No** |
| ^b^AST (U/L) | **21.0** | **18.0** | ^b^*CD117* | **(+ve)** | **(-ve)** | ^b^*Obesity* | **No** |
| ^b^Bilirubin (mg/dL) | **0.4** | **0.4** | ^b^*CD11B* | **(-ve)** | **(-ve)** | *AML type* | **Primary** |
| ^b^Calcium (mg/dL) | **7.9** | **9.3** | ^b^*CD123* | **(-ve)** | **(-ve)** | *Non-AML cancer* | **No** |
| ^b^Creatinine (mg/dL) | **0.9** | **0.9** | ^b^*CD13* | **(+ve)** | **(-ve)** | *Treatment related AML* | **Yes** |
| ^b^Fibrinogen (mg/dL) | **638.0** | **NA** | ^b^*CD14* | **(-ve)** | **(-ve)** |  |  |
| ^b^Hemoglobin (g/dL) | **8.5** | **9.2** | ^b^*CD15* | **(-ve)** | **(-ve)** |  |  |
| ^b^LDH (U/L) | **235.0** | **169.0** | ^b^*CD33* | **(+ve)** | **(-ve)** |  |  |
| ^b^Lymphocytes (10^9^/L) | **0.5** | **0.5** | ^b^*CD34* | **(+ve)** | **(-ve)** |  |  |
| ^b^Phosphorus (mg/dL) | **2.1** | **4.3** | ^b^*CD38* | **(+ve)** | **(-ve)** |  |  |
| ^b^Platelets (10^9^/L) | **130.0** | **88.0** | ^b^*CD45* | **(+ve)** | **(-ve)** |  |  |
| ^b^Potassium (mmol/L) | **3.7** | **4.0** | ^b^*CD56* | **(-ve)** | **(-ve)** |  |  |
| ^b^Uric-acid (mg/dL) | **2.6** | **3.0** | ^b^*CD64* | **(-ve)** | **(-ve)** |  |  |
| ^b^WBC (10^9^/L) | **0.4** | **2.7** | ^b^*HLADR* | **(+ve)** | **(-ve)** |  |  |
|  |  |  | ^b^*MPO* | **(+ve)** | **(-ve)** |  |  |
|  |  |  | ^b^*EGR1* | **(-ve)** | **(NP)** |  |  |
|  |  |  | ^b^*MLL* | **(-ve)** | **(NP)** |  |  |
|  |  |  | ^b^*7Centromere* | **(-ve)** | **(NP)** |  |  |
|  |  |  | ^b^*8Centromere* | **(-ve)** | **(NP)** |  |  |
|  |  |  | ^b^*Cytogenetics* | **Intermediate** | **Intermediate** |  |  |
|  |  |  | *ELN risk* | **Adverse** | **Adverse** |  |  |
|  |  |  | ^b^*CBFB* | **(-ve)** | **(NP)** |  |  |
|  |  |  | ^b^*FLT3* | **(-ve)** | **(NP)** |  |  |
|  |  |  | ^b^*IDH2* | **(-ve)** | **(NP)** |  |  |
|  |  |  | ^b^*NPM1* | **(-ve)** | **(NP)** |  |  |
|  |  |  | ^b^*RUNX* | **(-ve)** | **(NP)** |  |  |

**7+3**

| **TX related side effects, t_<30_** | |
| --- | --- |
| ^b^Creatinine toxicity (CTCAE) | **Non-toxic** |
| ^b^CKD (CTCAE) | **Unknown** |
| ^b^Bilirubin toxicity (CTCAE) | **Non-toxic** |
| ^b^AST toxicity (CTCAE) | **Non-toxic** |
| ^b^ALT toxicity (CTCAE) | **Non-toxic** |
| ^b^Proteinuria (CTCAE) | **Non-toxic** |
| ^b^Ejection fraction (CTCAE) | **G2** |
| ^b^Anemia toxicity (CTCAE) | **G3** |
| ^b^Thrombocytopenia (CTCAE) | **G4** |
| ^b^Neutrophils (CTCAE) | **G4** |
| ^b^Febrile neutropenia toxicity (CTCAE) | **G3** |

| **Responses by 30d FUP Bx date, t_<30_** | |
| --- | --- |
| ^b^FUP Response at 30d | **CRi** |
| ^b^Difference between TX start date & 30d FUP Bx date | **(30,55]** |
| ^b^Hospital re-admission | **No** |
| ^b^Hospital ICU re-transfer | **No** |
| ^b^RBC transfusions (bags) | **0** |
| ^b^Platelet transfusions (bags) | **0** |
| Stem-cell transplant | **No** |

| **Long term responses** | |
| --- | --- |
| Survival status | **Alive** |
| Time-to-censor (days) | **1002** |

| **Induction related responses, t_ind_** | |
| --- | --- |
| ^b^ICU transfer | **No** |
| ^b^Hospitalization (days) | **25** |
| ^b^RBC transfusions (bags) | **6** |
| ^b^Platelet transfusions (bags) | **4** |


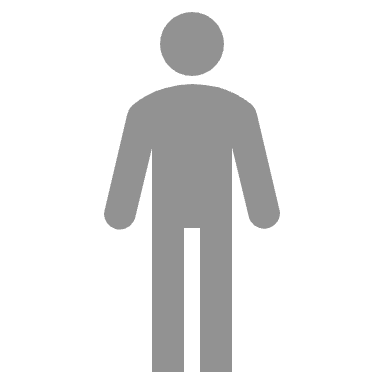

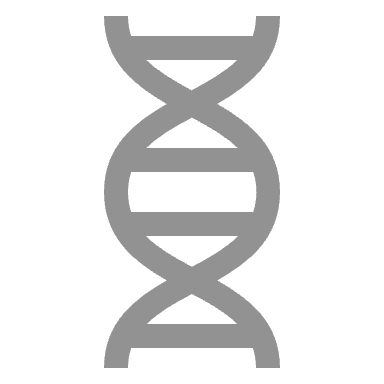

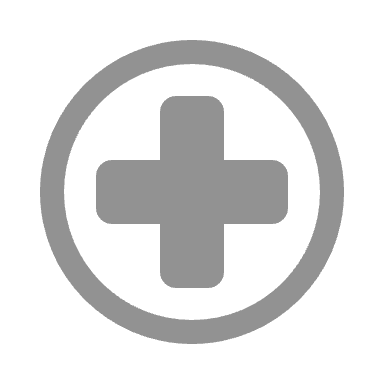

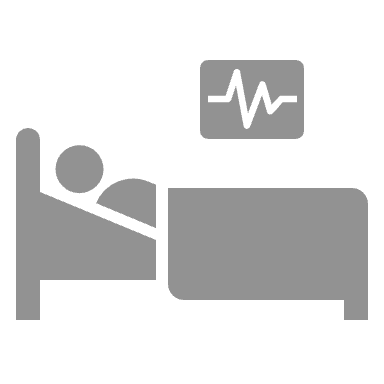

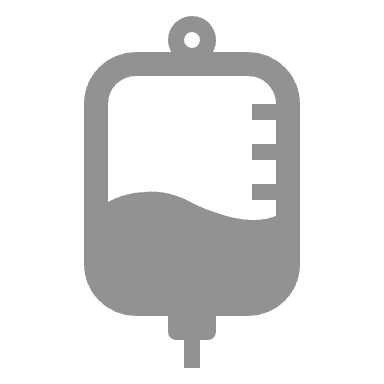

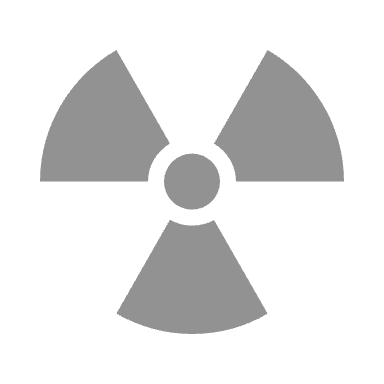

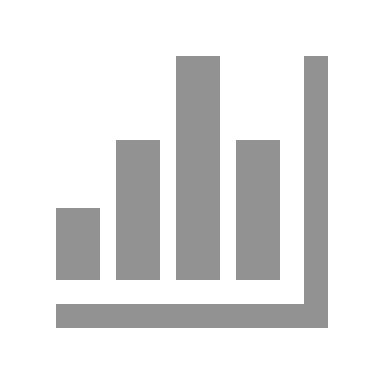


Remark: ^b^Features used in ML model

**Overall survival up to 2 years**

| **7+3** | **Venetoclax + Azacitidine** |
| --- | --- |

**KM probability at (365d,730d) = (0.82,0.67)**

**Training model cAUC: 1.00 || C_365_: 0.89 || Brier_365_: 0.03**

**KM probability at (365d,730d) = (0.55,0.35)**

**Training model cAUC: 0.96 || C_365_: 0.87 || Brier_365_: 0.13**


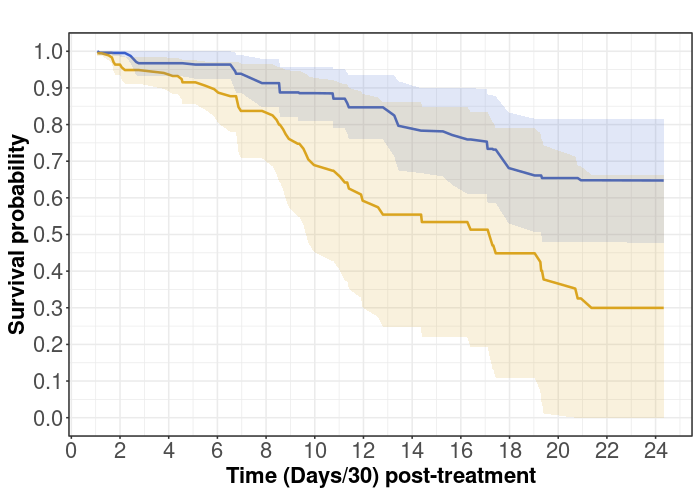

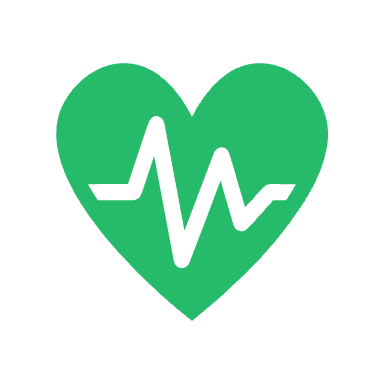


**Supplemental Figure 8. Disease state transitions during the first year of treatment: 7+3 (left) vs ven/aza (right).** Width of an arrow represents the frequency of interstate transitions.


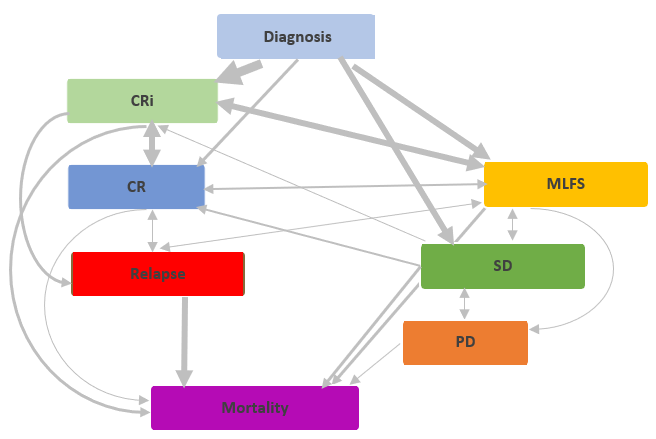


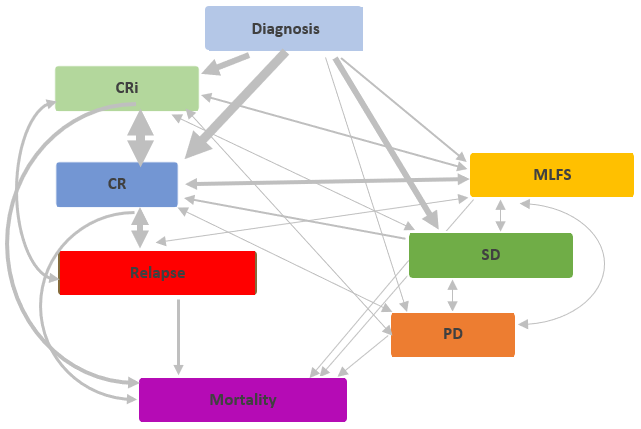


**Supplemental Figure 9. Disease state transition probabilities between days 90-365: 7+3 (top) vs. ven/aza (bottom).**

**To**

**From**

**To**

**To**

**From**

**To**


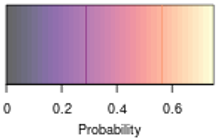


**0.0**

**0.20**

**0.40**

**0.60**

**0.75**

**Probability**

**7+3**


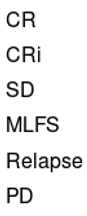

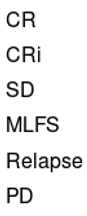

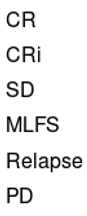

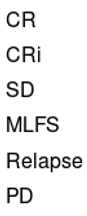

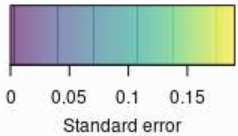


**(t_90,_ t_365_)**

**Ven/**

**Aza**

**0.0**

**0.05**

**0.10**

**0.15**

**0.25**

**Standard error**


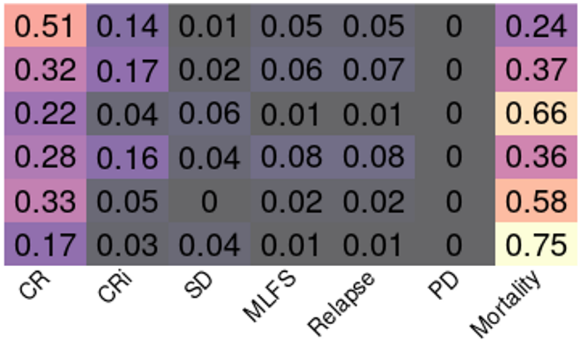

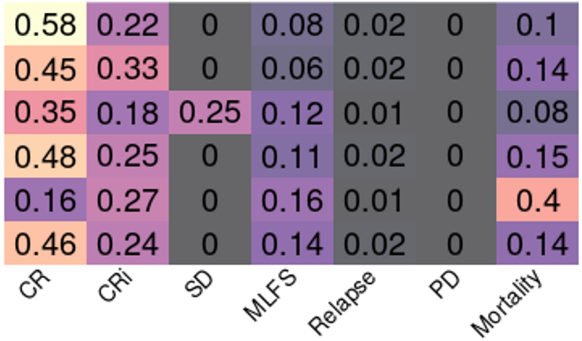

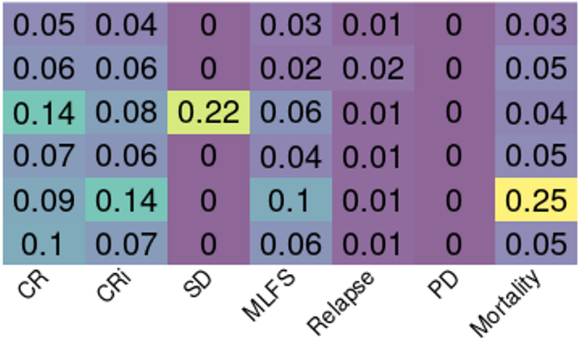

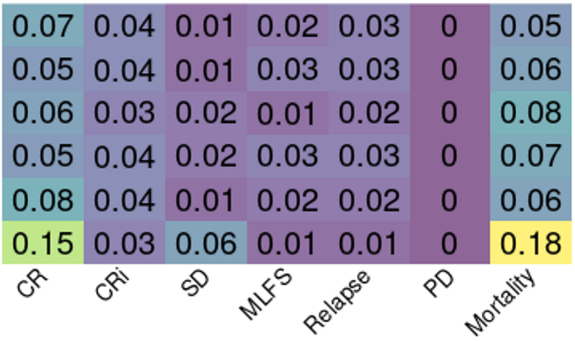


**Supplemental Figure 10. Disease state transitions probabilities in days 180-365: 7+3 (top) vs. ven/aza (bottom).**

**To**

**From**

**To**

**To**

**From**

**To**

**0.0**

**0.2**

**0.4**

**0.6**

**0.8**

**Probability**

**7+3**


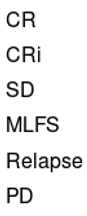

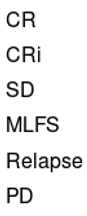

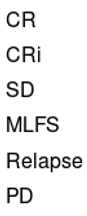

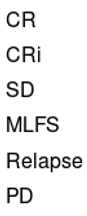


**(t_180,_ t_365_)**

**Ven/**

**Aza**

**0.0**

**0.10**

**0.15**

**0.20**

**0.35**

**Standard error**


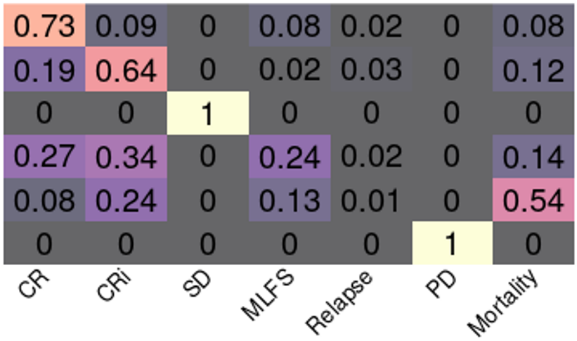

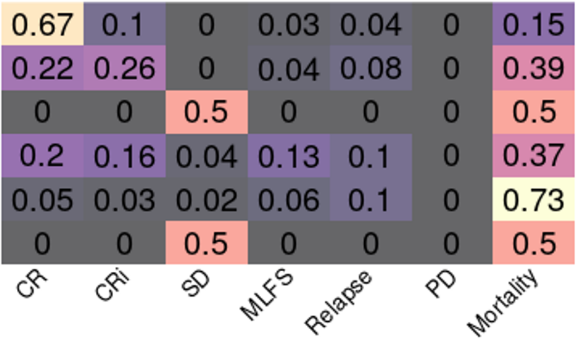

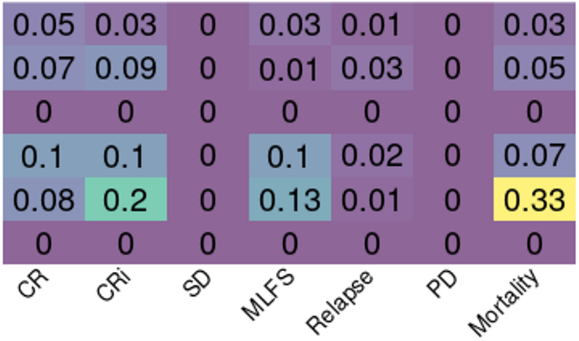

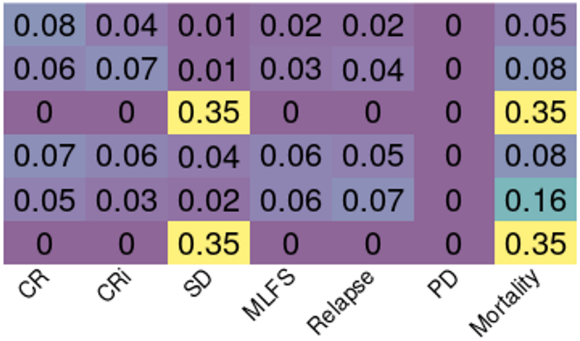

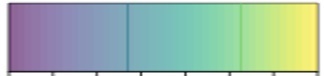

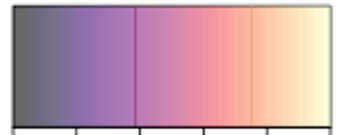


**1.0**
